# Data-driven treatment selection for seamless phase II/III trials incorporating early-outcome data

**DOI:** 10.1002/pst.1619

**Published:** 2014-05-02

**Authors:** Cornelia Ursula Kunz, Tim Friede, Nick Parsons, Susan Todd, Nigel Stallard

**Affiliations:** a Warwick Medical School, The University of WarwickCoventry CV4 7AL, UK; b Department of Medical Statistics, University Medical Center GöttingenGöttingen, Germany; c DZHK (German Centre for Cardiovascular Research), Partner Site GöttingenGöttingen, Germany; d Department of Mathematics and Statistics, University of ReadingReading RG6 6AX, UK

**Keywords:** adaptive seamless design, multi-arm multi-stage trial, surrogate endpoint

## Abstract

Seamless phase II/III clinical trials are conducted in two stages with treatment selection at the first stage. In the first stage, patients are randomized to a control or one of *k* > 1 experimental treatments. At the end of this stage, interim data are analysed, and a decision is made concerning which experimental treatment should continue to the second stage. If the primary endpoint is observable only after some period of follow-up, at the interim analysis data may be available on some early outcome on a larger number of patients than those for whom the primary endpoint is available. These early endpoint data can thus be used for treatment selection. For two previously proposed approaches, the power has been shown to be greater for one or other method depending on the true treatment effects and correlations. We propose a new approach that builds on the previously proposed approaches and uses data available at the interim analysis to estimate these parameters and then, on the basis of these estimates, chooses the treatment selection method with the highest probability of correctly selecting the most effective treatment. This method is shown to perform well compared with the two previously described methods for a wide range of true parameter values. In most cases, the performance of the new method is either similar to or, in some cases, better than either of the two previously proposed methods. © 2014 The Authors. *Pharmaceutical Statistics* published by John Wiley & Sons Ltd.

## 1. INTRODUCTION

Drug development is very expensive and risky with many compounds failing in late development phases. Adaptive designs have been recognized as a way to improve efficiency of drug development by industry and regulators alike 1–3. Of particular interest are designs combining aspects of the clinical development process into one single study that would have traditionally been assessed in separate trials and phases, for instance, adaptive seamless phase II/III designs 4–7. Seamless phase II/III clinical trials are conducted in two stages. In the first stage, patients are randomized to control or some number *k* > 1 experimental treatments. At the end of this stage, interim data are analysed, and a decision is made concerning which experimental treatment should continue, along with the control, to the second stage. If the primary endpoint is observable only after some period of follow-up, at the interim analysis, data may be available on some early outcome on a larger number of patients than those for whom the primary endpoint is available. These early endpoint data can thus be used for guiding the choice of treatments to continue. It has been demonstrated, for a range of settings, that adaptive trial designs are generally more efficient, and thereby quicker due to improved resource management, than conventional programmes with phase II studies for treatment selection and phase III studies for confirmation of the efficacy of the selected treatments 8.

Two different procedures for incorporating early endpoint data in a treatment selection design have been proposed by Stallard 9 and Friede *et al.* 10; the focus in this paper is on treatment selection, general principles and methods for hypothesis testing in adaptive seamless designs are discussed in detail elsewhere 8,5,10, as are alternative methods in the setting where early endpoint data are available at interim analysis 11,12. Friede *et al.* propose a method of treatment selection using early endpoint data only. In contrast, Stallard uses any available final (primary) endpoint data in addition to early endpoint data for treatment selection at interim. Although both approaches differ in the way in which data from the two stages are combined, they have both been shown to control the type I error rate. In this paper, we propose a new data-driven methodology to pick the optimal approach to treatment selection, which uses data available at an interim analysis to estimate treatment effects and correlations between endpoints and then, on the basis of these estimates, chooses the treatment selection method with the highest probability of selecting the most effective treatment. This new data-driven method is compared with the more established data-driven methods of Friede *et al.* and Stallard for a wide range of true parameter values. We focus here on using interim data available at an early point in a trial to select the method to undertake treatment selection. If, however, this was not possible, and it was necessary to decide on the appropriate method at the planning stage, then one would have little option but to rely on for instance other (external) sources of information or data (possibly from a pilot study) to augment ones beliefs about the likely effect sizes and associations between early and final endpoints. Section 2 describes a randomized trial assessing the efficacy of a novel compound in patients with primary hypertension, which provides a motivating example of how early outcome data might be used for decision-making at an interim analysis of a seamless phase II/III trial. Appropriate notation is established, and treatment selection for the two previously proposed methods is described in detail in Section 3. The new data-driven selection rule is developed in Section 4. Section 5 provides a worked example of how selection probabilities are calculated using data from the motivating example. In Section 6, numerical evaluation of the new data-driven selection rule is undertaken in a simulation study, and the performance of the new method is compared with the two previously proposed methods. The paper concludes with a discussion in Section 7.

## 2. MOTIVATING EXAMPLE

**Figure 1:**
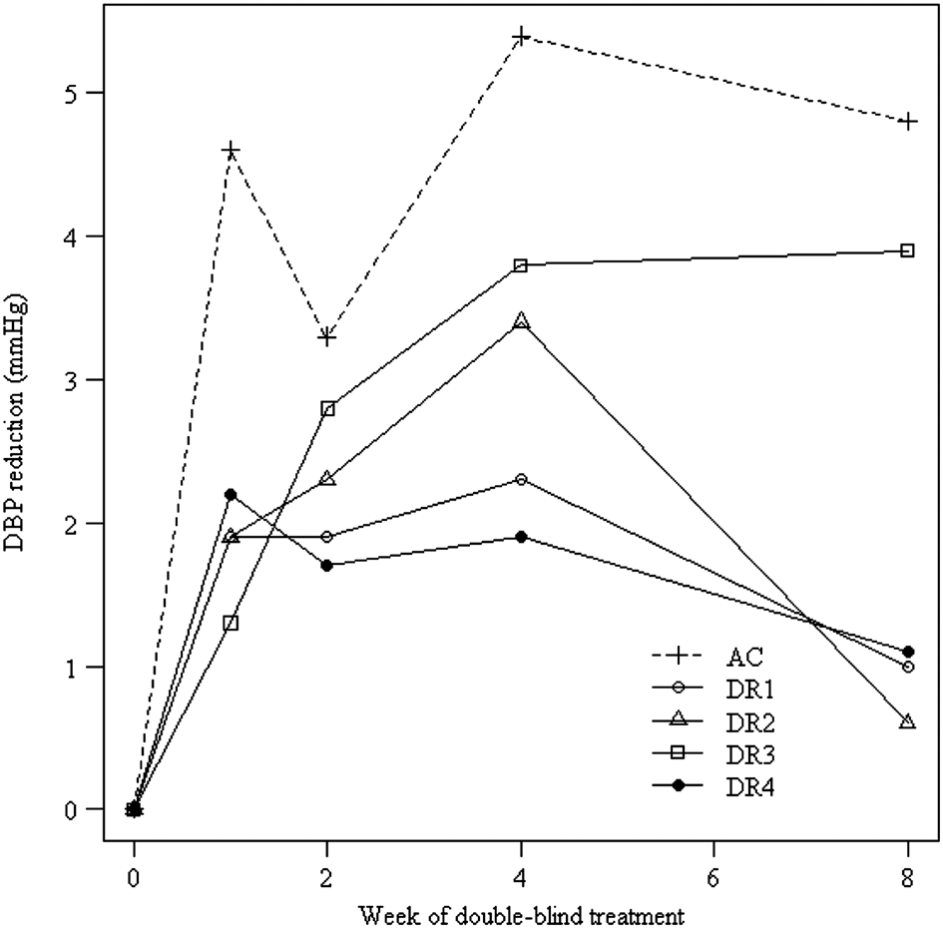
Differences in diastolic blood pressure (DBP) between active treatments (i.e. dose regimens (DR) 1-4 and active control (AC)) and placebo over the time course of the 8 weeks of double-blind treatment.

Calhoun *et al.* 13 report a double-blind, placebo-controlled, randomized trial assessing the efficacy and safety of a novel compound in patients with primary hypertension. The primary efficacy endpoint was the change in clinic diastolic blood pressure (DBP; mmHg) from baseline to the end of the 8-week double-blind treatment period. A total of n=524 patients were randomized to receive either one of four dose regimens of the new anti-hypertensive, an active control (AC) or placebo for 8 weeks. The standard deviation (SD) of the differences from baseline was assumed to be 10 mmHg in the sample size calculation. Additional readouts of the primary endpoints were available at weeks 1, 2 and 4. The primary comparisons were with placebo (and not with the AC) using a multiple-contrast test 14 to control the familywise type I error rate at 0.025 one-sided. Figure 1 gives the differences in diastolic blood pressure (DBP) between the dose regimens and AC with placebo over the time course of the 8 weeks of double-blind treatment. The differences of dose regimens 3 (DR3) and the AC to placebo at the end of the 8-week treatment period were found to be statistically significant. These two treatments also showed the biggest reductions in DBP at weeks 2 and 4 in comparison to placebo. Here, we investigate the possibility that a single interim analysis be undertaken at some point before the trial endpoint had been observed on all patients, but where, dependent on timing, a single early endpoint were available on a group of patients, and additionally some primary efficacy endpoint data were also available from a sub-group of these patients at interim. We would expect both that differences in DBP at different time points for the same patient would be correlated and that treatment effects on the earlier outcomes might indicate the ordering of treatment effects for the final outcome. This suggests that we might be able to use the early time point data in the treatment selection at an interim analysis. The patients were recruited into the trial over a period of about seven months. Since the trial included a two-week washout and a two-week single-blind run-in period, at an interim analysis half way through recruitment, say after 4 months, only those patients recruited during the first month would have had completed their 8-week double-blind treatment period whereas the other patients could have only contributed 1-, 2- or 4-week measurements to the interim analysis. In what follows, we will explore selection rules for adaptive treatment selection at interim incorporating incomplete patient follow-up data.

## 3. PHASE II/III CLINICAL TRIALS WITH EARLY OUTCOME DATA

### 3.1. A flexible hypothesis testing method for phase II/III clinical trials with early outcome data

Following, e.g. Stallard 9, we envisage a two-stage clinical trial, in which in the first stage, patients are randomized to the control treatment *T*_0_ or to one of *k* experimental treatments, *T*_*i*_,*i* = 1, … ,*k*, with one of these experimental treatments, *T*_*I*_, selected to continue, along with the control treatment, *T*_0_, to the second stage.

We suppose that, at the end of the first stage, data are available on the primary, final outcome for *n*_1_ patients in each treatment group, but that in addition, early outcome data are available for some larger number of patients, *N*_1_ per group. At the end of the second stage, final outcome data will be available for all *n*_2_ patients receiving treatments *T*_*I*_ and *T*_0_.

Denote by *X*_*i*,*j*_ and *Y*_*i*,*j*_ respectively the early and final outcome data from patient *j* in group *i*,* i* = 0, … ,*k*,* j* = 1, … ,*n*_2_. The two endpoints for each patient are assumed to follow a bivariate normal distribution with

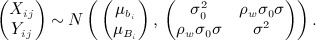
(1) Given the mean values, individual patients are assumed to be independent so that 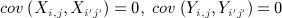 and 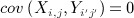 for *i* ≠ *i* ′  or *j* ≠ *j* ′ .

The parameters of interest are the treatment effects on the final outcome for each treatment relative to the control, 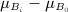, which will be denoted by *θ*_*i*_,* i* = 1, … ,*k*, with *H*_0*i*_ denoting the null hypotheses *θ*_*i*_ = 0, which will be tested against the one-sided alternative hypotheses *H*_1*i*_ : *θ*_*i*_ > 0. The familywise type I error rate may be controlled in the strong sense by application of the closed testing procedure 15 and combination test 16,17. Intersection hypotheses 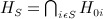, where *S* ⊆ {1, … ,*k*}, are tested using data from each of the two stages and combined using a combination test. Individual elementary hypotheses *H*_0*i*_ are rejected at level *α* if and only if all intersection hypotheses with index sets that include *i* are rejected at individual local test level *α* 8. Stagewise *p*-values, *p*_*S*,1_ and *p*_*S*,2_, are combined using the weighted inverse normal method, with (pre-specified) weights (*w*_1_ and *w*_2_) proportional to the planned sample size at each stage, with *H*_*S*_ rejected at level *α* if *C* (*p*_*S*,1_,*p*_*S*,2_) ≤ *α* where *C* (*p*_*S*,1_,*p*_*S*,2_) = 1 − Φ (*w*_1_Φ^ − 1^ (1 − *p*_*S*,1_) + *w*_2_Φ^ − 1^ (1 − *p*_*S*,2_)) 18.

For *σ* assumed known, a test of *H*_0*i*_ may be based on standardized test statistics using final endpoint data from stages one and two given by

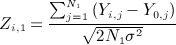

and

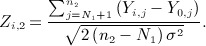


Note that, in order to ensure independence between test statistics from the two stages, *Z*_*i*,1_ uses data from all patients for whom early endpoint data are available in stage one and *Z*_*i*,2_ uses data from only those patients recruited in the second stage. From equation (1), *Z*_*i*,*j*_ are normally distributed, with 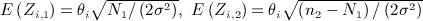, 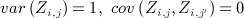 for *j* ≠ *j* ′  and 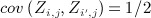 for *i* ≠ *i* ′ . The *p*-values for a test of each of the intersection hypotheses *H*_*S*_ at each stage may be obtained by a Dunnett test 19 that uses the test statistic *Z*^*max*^ = max_*i* ∈ *S*_*Z*_*i*,*j*_. The second stage *p*-value for a test of *H*_*S*_,* p*_*S*,2_, is set to *p*_*I*_, the *p*-value for selected treatment *T*_*I*_, if *I* ∈ *S* and to 1 if *I* ∉ *S*, as in the conservative approach suggested by Posch *et al.* 20.

This approach to hypothesis testing in the setting of treatment selection with early endpoint data was proposed by Friede *et al.* 10 who considered specifically the setting where early endpoint data only were available for decision making (treatment selection) at stage one. Although they also propose a treatment selection rule for use in this setting, the analysis approach controls the familywise type I error rate in the strong sense for any treatment selection method based on the data observed at the end of stage one. An alternative treatment selection method for a setting in which a combination of final and early endpoint data are available from stage one was proposed by Stallard 9. The treatment selection methods of Friede *et al.* and Stallard are described in detail in the succeeding text.

### 3.2. Treatment selection method of Friede *et al.*

Friede *et al.* 10 proposed a method for selection of the treatment *T*_*I*_ that should continue to the second stage. In the setting they envisage, early endpoint data only are available at the time when the treatment selection is made, so that the selection does not use any final endpoint data. The method can, however, be applied when some final endpoint data are available, as discussed by Kunz *et al.* 21.

Let

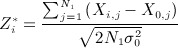

be the standardized test statistic based on the early endpoint data from stage one. From equation (1), 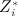 are normally distributed with 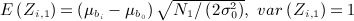 and 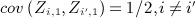.

Treatment *T*_*I*_ is then selected where 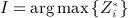. The probability that, e.g. treatment 1 is selected is therefore given by

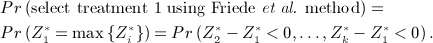


This may be calculated using the joint distribution of 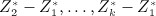 given by

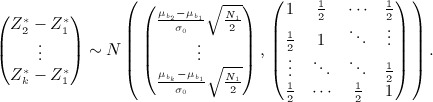


Denoting by *F*(**x**,*μ*,**Σ**), the cumulative distribution function of a multivariate normal with mean *μ* and variance-covariance matrix **Σ** evaluated at **x**, we have

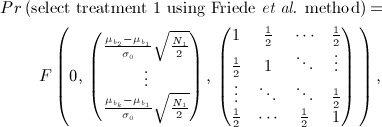
7 where here **0** denotes the zero-vector with *k* − 1 elements. Similar joint distributions enable calculation of the probabilities that other treatments are selected.

### 3.3. Treatment selection method of Stallard

Stallard 9 proposed a method for selection of the treatment *T*_*I*_ that should continue to the second stage using a combination of early and final endpoint data.

Let *S*_*i*_ denote the standardized score statistic for *θ*_*i*_ given all data available at the end of stage one. For known *σ*,* σ*_0_ and *ρ*_*w*_,* S*_*i*_ is given by

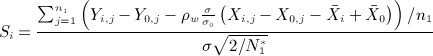

where 
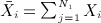
 and 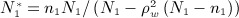. With *σ*,* σ*_0_ and *ρ*_*w*_ unknown, *S*_*i*_ may be estimated using the double regression method of Engel and Walstra 22; Galbraith and Marschner 23 provide an analogous expression to the above in the setting of a series of interim analyses at which the two groups are compared, with the number of patients for whom short-term and long-term data are available increasing through the duration of the trial. Stallard and Kunz *et al.* show that *S*_1_, … ,*S*_*k*_ follow a multivariate normal distribution with 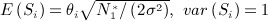 and 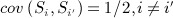.

Stallard proposes selecting treatment *T*_*I*_ where *I* = arg max{*S*_*i*_}. The probability that, e.g. treatment 1 is selected is therefore given by

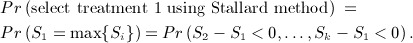


This may be calculated using the joint distribution of *S*_2_ − *S*_1_, … ,*S*_*k*_ − *S*_1_ given by

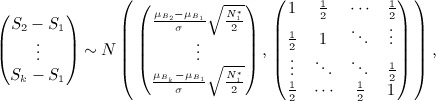

so that

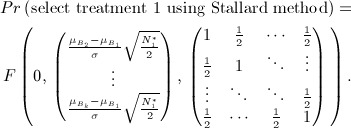
11 As in the preceding text, similar joint distributions enable calculation of the probabilities that other treatments are selected.

### 3.4. Practical considerations

Successful application of the methods of Friede *et al.* 10 and Stallard 9, described in Sections 3.2 and 3.3, depends in part at least on the adequacy of the early endpoint as a surrogate for the final endpoint. In a trial setting where a group of experimental treatments (i.e. two or more) are compared with a control the concept of group-level surrogacy corresponds to correlation between the group means for the early (surrogate) and final endpoints, and analogously individual level surrogacy corresponds to within-group correlation. For a fully validated surrogate endpoint, we require both individual and group-level surrogacy. For the methods of both Friede *et al.* and Stallard, the use of an early endpoint for decision making improves the power of the test. However, the properties of the two methods are very different. For the method of Stallard, the gain in power comes from the within-group correlation between the early (surrogate) and final outcomes (*ρ*_*w*_); i.e. from the presence of individual-level surrogacy. Whereas for the method of Friede *et al.* unless there is a large within-group correlation, the gain in power arises from the correlation between treatment effects for the early and final outcomes; i.e. from the presence of group-level surrogacy. The latter property implies that in some settings, the Friede *et al.* method could perform badly. For instance, consider a setting where the 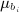
's were widely spaced, but the 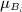
's were close together. The method of Friede *et al.* will pick a treatment based on early outcome data 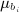 with high probability, but because of the close spacing of the 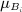
's, it is likely that the selected treatment may not perform equally well based on the final outcome. Clearly, the Friede *et al.* methodology is only really sensible if the early endpoint data alone provide useful information for treatment selection; if it leads one to pick the *wrong* treatment(s), it is not sensible. The Stallard method may do better in such settings, dependent on the magnitude of *ρ*_*w*_. So we would advise that before either of these methods are used, some thought is given to the issues discussed here, and the likely nature and magnitude of all associations between early and final outcomes be considered during the trial planning stage. Kunz *et al.* 21 provide a comparison of the treatment selection rules proposed by Stallard and Friede *et al.* They show that the properties of the methods depend on the true unknown treatment effects and correlations between the endpoints, and that neither the Stallard method nor the Friede *et al.* method is always preferable in terms of selection probability or power. The new data-driven selection rule discussed in Section 4 describes how one might select which of the two methods to use, but if one had strong beliefs or data to support one or other methodology above the other at the planning stage of a trial, then clearly the most sensible option would be to proceed with that methodology.

## 4. A NEW DATA-DRIVEN SELECTION RULE

In this section, we introduce a new treatment selection method based on the methods proposed by Friede *et al.* and Stallard. The idea is that following observation of the data at the end of stage one, both the methods of Friede *et al.* and Stallard will be applied and compared. If both methods agree on which treatment should proceed along with the control to the second stage, the treatment indicated by both methods will be selected. If the methods indicate that different treatments should proceed, the stage one data will be used to assess which of the methods is likely to be correct, as explained in more detail in the remainder of this section, and the treatment indicated by this method will be selected.

The aim is to provide a method that has high power, where this is defined to be the probability of correctly selecting the most effective treatment and rejecting the null hypothesis corresponding to this treatment having effect no greater than the control treatment. This probability is bounded above by the probability that the most effective treatment is selected on the basis of the data observed at stage one. In principle, it may be possible to compute the power analytically; however, it is very hard compared with computation of the selection probabilities from (7) and (11); our proposed method for choosing between the treatment selection rules is based on the latter method.

Given observed stage one data, estimates of the effects, *μ*_*b*_ and *μ*_*B*_, variances, *σ*_0_ and *σ*, and the correlation, *ρ*_*w*_, can be obtained. These will be denoted by 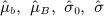 and 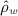, respectively.

On the basis of these estimates, we can calculate the estimated probability of selection of each treatment for each of the two methods given by (7) and (11), with the estimated probability of, e.g. selection of treatment *T*_1_ given by

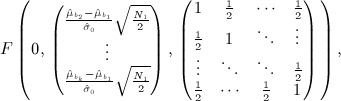
12 for the Friede *et al.* selection method and

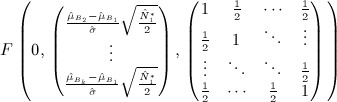
13 for the Stallard selection method, where 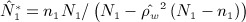.

These estimated selection probabilities give an indication of the preference of each treatment based on each selection method. For the Friede *et al.* and Stallard selection methods, the treatments with the largest estimated selection probability is that with the largest value of 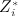 and *S*_*i*_, respectively, and so will be the treatments selected. When the two methods indicate selection of different treatments, we therefore propose to select the method for which the estimated selection probability for the selected treatment is largest. With the treatment selected in this way, we propose to test the hypotheses *H*_0*i*_,*i* = 1, … ,*k* using the method as described in Section 3.1. As Friede *et al.* show that this controls the familywise type I error rate in the strong sense for any treatment selection method based on data from patients enrolled in stage one of the trial, the familywise type I error rate is strongly controlled.

## 5. EXAMPLE

The motivating example provides an informative setting in which to demonstrate how selection probabilities are calculated for the Stallard and Friede *et al.* methods. Let us consider the possibility that an interim analysis were undertaken where early outcome data, DBP at the 4-week assessment time point, were available from *N*_1_ patients, and 8-week assessment data, the primary efficacy endpoint, were available from *n*_1_ patients. For the selected motivating example, we would expect there to be strong within group correlations (*ρ*_*w*_) between early (4-week) and final (8-week) outcome data. Also, available data (Figure 1) suggest strong correlations between treatment group effects, albeit the sample of available treatment group means is small. Clearly, in this setting, the methods of both Friede *et al.* and Stallard have potential application, so it is of interest to understand under what conditions one or other method performs the better. Depending on the timing of the interim analysis, we investigate two options; (i) where 4 week data were available from half of the target population (*N*_1_ = 45), and 8 week data were also available from a small proportion (*n*_1_ = 10) of these patients, and (ii) where the interim analysis was undertaken at an earlier time point where *N*_1_ = 25 and *n*_1_ = 5. The AC treatment is always selected in addition to the placebo. The parameters of interest, the effects for the four test treatments, on the final outcome relative to the placebo 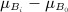 for *i* = 1, … ,4, are estimated from Figure 1 to be approximately 1.0, 0.6, 3.9 and 1.1 mmHg for treatments DR1, DR2, DR3 and DR4, respectively. Similarly, the estimates of early outcome (4 week) treatment effects relative to the placebo 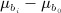 are estimated from Figure 1 to be approximately 2.3, 3.4, 3.8 and 1.9 mmHg. Given these estimates for 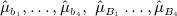, estimates for early and final outcome standard deviations of 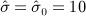 and correlation parameter 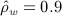, treatment selection probabilities for the Stallard and Friede *et al.* methods can be calculated using expressions (12) and (13). These can be evaluated simply using the pmvnorm function in the mvtnorm package 24 in R 25, to give treatment selection probabilities for setting (i) of (0.102, 0.072, 0.716, 0.110) and (0.118, 0.337, 0.468, 0.076) for the Stallard and Friede *et al.* methods respectively and for setting (ii) treatment selection probabilities of (0.143, 0.113, 0.592, 0.152) and (0.150, 0.324, 0.426, 0.110) for the two methods, all for treatments DR1, DR2, DR3 and DR4 respectively. Both methods would select treatment DR3 with highest probability, in both settings, with setting (ii), where fewer patients were available, having lower selection probabilities for the DR3 group. Treatment group DR2 illustrates where the methods can differ. For the Stallard method the selection probability for this group is much smaller than for the Friede *et al.* method, due to the relatively poor performance of this treatment group at the 8-week assessment, from which data are not used by the Friede *et al.* method for determining selection probabilities. In general, the two methods may give different selection probabilities, the properties of these procedures together with the data-driven method are explored in more detail in a simulation study.

## 6. NUMERICAL EVALUATION OF THE NEW DATA-DRIVEN SELECTION RULE

### 6.1. Selection probability and power

In general, there are two different ways to define the selection probability: (i) the probability to select any effective treatment and (ii) the probability to select the most effective treatment. Throughout this paper, we use the latter definition. In order to be consistent with this definition, we also define the power as the probability to reject the null hypothesis belonging to the most effective treatment. Furthermore, we will, without loss of generality, focus on *T*_1_ and report the probability of selecting *T*_1_ as well as rejecting the corresponding null hypothesis, *H*_01_.

In this section, we report the results of a simulation study to explore the properties of the new procedure. Results are shown for a trial with three experimental groups, that is *k* = 3, compared with a control group, *T*_0_, with *n*_1_ = 4,* N*_1_ = 32 and *n*_2_ = 64. For treatment *T*_1_, we assume final endpoint effects, 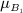, from − 0.5 to 1 in steps of 0.1 and early endpoint effects, 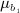, of -0.2, 0, and 0.2, and we set 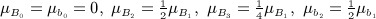 and 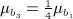 to give early and final endpoint effects of a half and a quarter of the size of the effect for *T*_1_. Note that even for 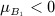, the probability to select *T*_1_ is reported although, in this case, this is the worst-performing treatment. We investigate correlation *ρ*_*w*_ values of − 0.9, − 0.5, 0, 0.5 and 0.9. Results are based on 10,000 simulations for each scenario considered. Supplementary material describes the results of additional simulation studies, covering a wide range of alternative parameter settings.

**Figure 2:**
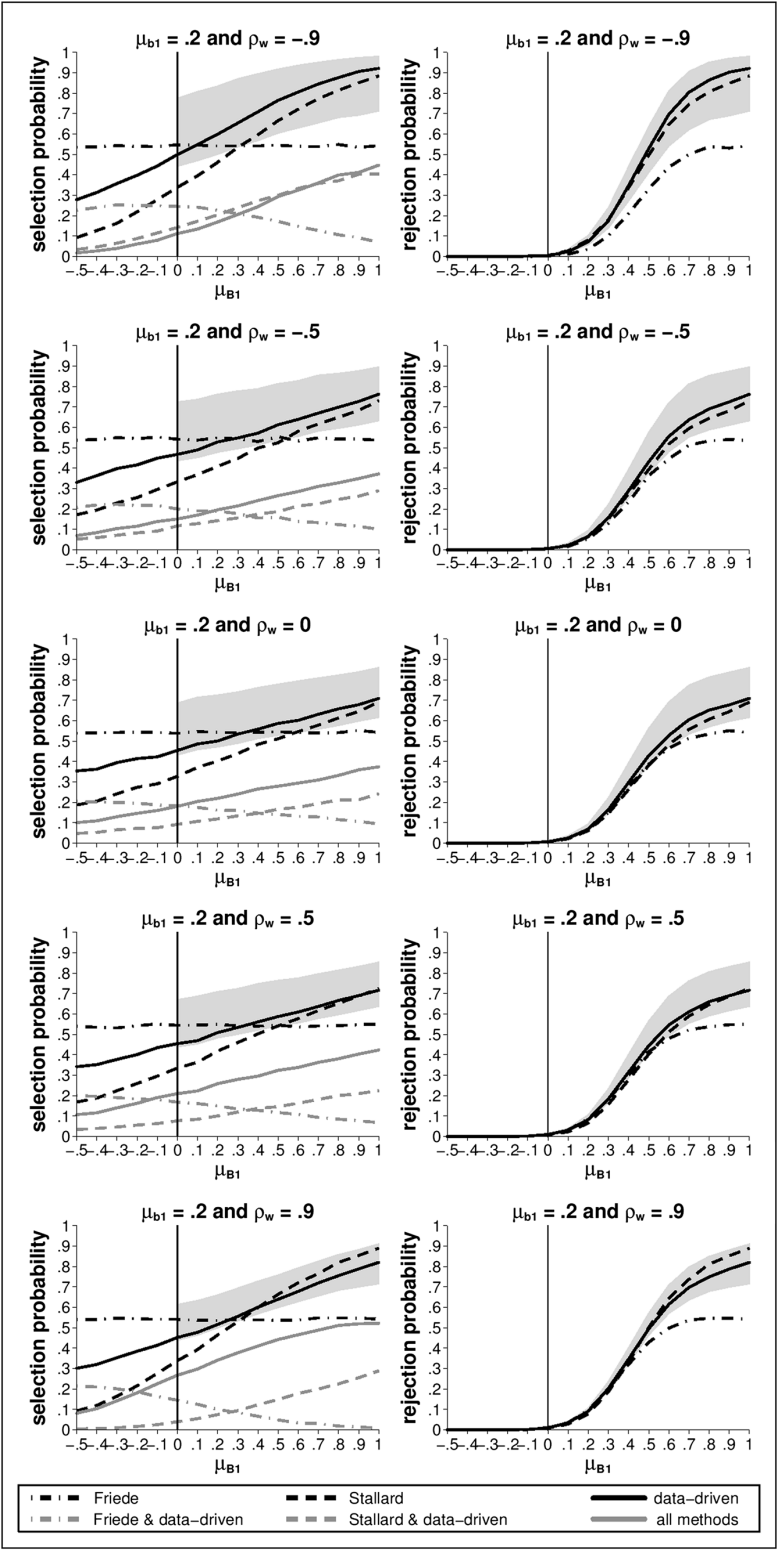
Selection (left) and rejection probabilities (right) for *T*_1_, as a function of final endpoint effect *μ*_*B*1_, using the Stallard 9, Friede et al. 10 and data-driven methods for *T*_1_ early endpoint effect *μ*_*b*1_ = 0.2 and correlation between endpoints within each group *ρ*_*w*_ = { − 0.9, − 0.5,0,0.5,0.9}; 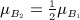, 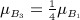, 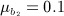 and 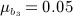.

Simulation results are shown in Figure 2. The panels on the left-hand side of the figure show the probability of selecting treatment *T*_1_ for the Stallard (black dashed line), the Friede *et al.* (black dash-dotted line) and the data-driven method (black solid line). The panels on the right-hand side show the probability of both selecting treatment *T*_1_ and rejecting *H*_01_ at the *α* = 0.025 level, which is the power as defined in the preceding text, for the three methods using the same line types.

For the Friede *et al.* method, the selection is based on early endpoint data only. The selection probability for this method thus does not depend on 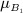 or *ρ*_*w*_. This is in contrast to the selection probability for the Stallard method, which increases with increasing 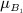 and, to a lesser extent, with the magnitude of *ρ*_*w*_.

For *ρ*_*w*_ positive, for 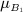 larger than about 0.3, the selection probability for the data-driven method is mostly similar to that for the Stallard method, i.e. the better of the two methods under these scenarios. For 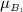 less than 0.3, the selection probability for the new method is between that for the two previous methods. For *ρ*_*w*_ less than zero, for 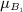 above about 0.3, the new method leads to selection of treatment *T*_1_ with higher probability than either the Stallard or Friede *et al.* methods.

Under the scenarios considered, the power is generally higher for the Stallard selection method than for the Friede *et al.* method. The power for the new method is generally similar to that for the Stallard method for larger *ρ*_*w*_ and greater than that for the Stallard method for smaller or negative *ρ*_*w*_.

In order to evaluate the performance of the data-driven method, two boundaries can be defined against which the data-driven method is then compared. The data-driven method can only select a treatment group that was selected by at least one of the Stallard and the Friede *et al.* method. An upper bound for the selection probability for the new method is thus the probability that at least one of the Stallard and Friede *et al.* methods selects treatment *T*_1_. This boundary is only meaningful if *T*_1_ is the most effective treatment, that is if 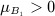. An alternative to choosing between the treatments selected by the Stallard and the Friede *et al.* methods using the data-driven rule introduced in this paper is to randomly select between the treatments recommended by the two methods when they disagree. The selection probability for such an approach would be the average of that for the Stallard and Friede *et al.* methods. Although not a lower bound for the selection probability, it is desirable that the new method should perform at least as well as this approach. The grey-shaded area in Figure 2 marks the range of probabilities bounded by these two values. Ideally, the results for the data-driven method should be between the boundaries and as close as possible to the upper one. For the selection probability, we see that the data-driven method starts closer to the lower boundary for smaller values of 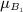 but comes closer to the upper boundary for higher values of 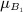. A range of power values defined similarly is shown on the plots on the right-hand side of Figure 2. The power for the data-driven method is quite close to the upper boundary for all scenarios considered.

To understand the results for the data-driven method in more detail, the selection probability can be split up in three different categories as illustrated by the grey lines on Figure 2: *T*_1_ was selected by the data-driven method because (1) the Stallard method but not the Friede *et al.* method selected *T*_1_ (grey dashed line), (2) the Friede *et al.* but not the Stallard method selected *T*_1_ (grey dash dotted line), (3) both the Friede *et al.* and the Stallard method selected *T*_1_ (grey solid line). Note that the probabilities for category (3) can be calculated using equation (14), given in the succeeding text and that the results for the three categories sum to the probabilities for the data-driven method. As explained above, it is more likely that both the Stallard and the Friede *et al.* methods select *T*_1_ if the correlation is positive than if the correlation is negative. For example, for 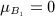, the probability that all three methods select *T*_1_ is just about 10% for *ρ*_*w*_ = − 0.9 but about 26% for *ρ*_*w*_ = + 0.9. On the other hand, the Stallard and the data-driven method selected *T*_1_ in about 14% of the cases for *ρ*_*w*_ = − 0.9 but in only 4% of the cases for *ρ*_*w*_ = + 0.9. Similarly, in about 25% of the cases, the Friede *et al*. and the data-driven method selected *T*_1_ if the correlation is negative, but in only 15% of the cases if the correlation is positive.

### 6.2. Effect of *ρ*_*w*_

It is interesting to note that although the selection probabilities and power when using the Stallard or Friede *et al.* selection method is the same for *ρ*_*w*_ of given magnitude, irrespective of the sign, the properties of the new data-driven selection method do depend on the sign of *ρ*_*w*_. The selection probabilities for the Friede *et al.* method and Stallard method are given by expressions such as equations (12) and (13), respectively. The first of these does not depend on *ρ*_*w*_, and the second depends on *ρ*_*w*_ only through 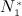, which depends on 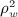, but not on the sign of *ρ*_*w*_. As the new selection method is based on the treatments indicated by both the Stallard method and the Friede *et al.* method, it is important to note that the treatments selected by the two methods are not independent, because they both depend on the early outcome data *X*_*i*,*j*_,*i* = 0, … ,*k*,*j* = 1, … ,*n*_1_. The probability that both methods lead to selection of treatment *T*_1_ is obtained from the joint distribution of 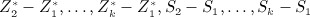, which does depend on the sign of *ρ*_*w*_, and is given by

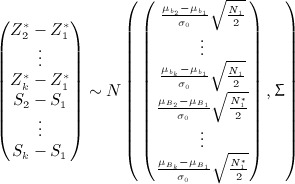
14 where

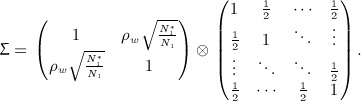


Joint distributions enabling calculation of the joint probability of selection of other treatments by the Stallard and Friede *et al.* methods are given by similar expressions.

The effect of the dependence on the sign of *ρ*_*w*_ is illustrated in Table 1, which gives the probabilities for each of the four cases that *T*_1_ is selected by (i) the Stallard method but not the Friede *et al.* method, (ii) the Friede *et al.* method but not the Stallard method, (iii) both methods, (iv) neither of the methods, together with the marginal distributions based on equations (7), (11) and (14). The first two sub-tables give probabilities for *ρ*_*w*_ = ± 0.9. For *ρ*_*w*_ = − 0.9, *T*_1_ is selected by both methods with probability 0.21 compared with 0.39 for *ρ*_*w*_ = 0.9. On the other hand, both methods fail to select *T*_1_ with probability only 0.13 for *ρ*_*w*_ = − 0.9 but with probability 0.31 for *ρ*_*w*_ = 0.9. Conversely, for *ρ*_*w*_ = − 0.9, *T*_1_ is selected by at least one of the two methods with probability 0.87 while for *ρ*_*w*_ = 0.9, *T*_1_ is selected by at least one of the two methods with probability 0.69. As the data-driven method can only select a treatment group that was selected by at least one of the Stallard and the Friede *et al.* method, the differences between the joint distributions for different signs of the correlation lead to the different results for the data-driven method that can be seen in Figure 2. An even more extreme example is given in the last two subtables in Table 1, when *ρ*_*w*_ = ± 1. In this case, when *ρ*_*w*_ = − 1, either the Stallard method or the Friede *et al.* method is correct but never both of them, while for *ρ*_*w*_ = 1, the two methods always agree, so that the probability of at least one method selecting treatment *T*_1_ changes from 0.33 to 0.67 depending on the sign of *ρ*_*w*_.

**Table 1:** Probability of selecting *T*_1_ for the Stallard and Friede *et al.* methods for (a) 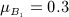, 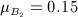, 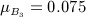, 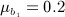, 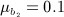 and 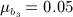, and (b) 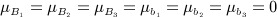 based on equations (7), (11) and (14).

		(a)						(b)					
		*ρ*_*w*_ = − 0.9			*ρ*_*w*_ = + 0.9			*ρ*_*w*_ = − 1			*ρ*_*w*_ = + 1		
		Stallard			Stallard			Stallard			Stallard		
		No	Yes	Total	No	Yes	Total	No	Yes	Total	No	Yes	Total
Friede	No	0.13	0.33	0.46	0.31	0.15	0.46	0.33	0.33	0.67	0.67	0.00	0.67
	Yes	0.33	0.21	0.54	0.15	0.39	0.54	0.33	0.00	0.33	0.00	0.33	0.33
Total		0.46	0.54	1.00	0.46	0.54	1.00	0.67	0.33	1.00	0.67	0.33	1.00

## 7. CONCLUSION

When a clinical trial with a primary endpoint that is observed only after a relatively long follow-up period includes interim analyses, it is desirable, if possible, to base these analyses at least in part on more rapidly observable short-term endpoint data. In general, any data available at the interim analysis could be used for treatment selection, as long as the type I error rate is controlled. Recently, Jenkins *et al.* 26 proposed a design for subgroup selection at interim using correlated survival outcomes. In this paper, we have considered two-stage adaptive seamless phase II/III clinical trials in which interim analysis data are used to decide which of a number of experimental treatments should continue along with the control treatment to the second stage. Two methods have previously been proposed for the use of short-term endpoint data in this setting 9,10. A comparison of the properties of these methods 21 has indicated that neither method is uniformly more powerful than the other, but that each can be more powerful depending on the (unknown) true values of the treatment effects on long-term and short-term endpoints and the correlation between the endpoints.

The method of analysis proposed by Friede *et al.* allows considerable flexibility in terms of the choice of the rule used to select the most promising treatment based on the interim data observed. In particular, the selection rule itself may be chosen in a data-dependent way based on data from patients recruited in stage one of the trial whilst maintaining control of the familywise type I error rate in the strong sense. This is the starting point for the method proposed in this paper, in which, when the Stallard and Friede *et al.* methods would lead to selection of different treatments, the method that maximizes the selection probability based on parameter values estimated from the observed data is used. The focus here has been on the development of a novel methodological approach, so for reasons of conciseness, we have not discussed some of the more practical issues concerning implementation, for instance recruitment patterns, whether recruitment should continue seamlessly and what triggers the interim analysis. These are all key issues, amongst many others, that would need to be considered when deciding whether the methods described here might work in any given setting; a fuller discussion of these issues is provided elsewhere 1,27. Mainly, for reasons of clarity of exposition, we have assumed fixed-effects models throughout this paper. Random-effects models for the methods of Stallard and Friede *et al.* have been discussed elsewhere 21, and also developed for the new data-driven approach of Section 4, but as the conclusions and simulation results were not changed qualitatively, the simpler fixed-effects approach only is presented here. We have restricted discussion in this paper to the most simple setting of a two-stage design where a single treatment is selected at an interim analysis, using a single early endpoint. Future work will investigate how this might be generalized to designs where more than one early endpoint were available for treatment selection, more than one treatment were selected at interim analysis and multiple (more than two) stages were planned.

The comparison in the preceding text, together with additional simulation results available as supplementary material, indicate that the new data-driven method has power higher than the average of the two existing methods, which is higher than a strategy that would pick one or other of the methods at random when they disagree, for nearly all parameter values considered. The only exceptions are when the treatment effect on the short-term endpoint for treatment 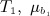, is less than 0 and the treatment effect for *T*_1_ on the long-term endpoint, 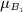 is close to 0, or when 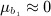 and *ρ*_*w*_ ≈ 1. In many cases, the new method is similar in performance to the most powerful of the other two methods. In some cases, the new method is better than either of the two exising methods, e.g. for large 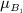, if *ρ*_*w*_ is close to 0 and 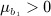, for nearly all values of 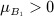, if *ρ*_*w*_ is close to − 1 and 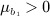 and for some 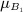 if *ρ*_*w*_ is close to 1 and 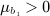.

The choice of whether or not to use the new data-driven method depends on the level of confidence in predictions of 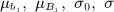 and *ρ*_*w*_ at the planning stage. If reliable estimates are available, it is possible to work out which method will give the best results based on equations (7), (11), and (14) and simulation studies for the power as reported above. Depending on the parameter estimates, as indicated above, the preferred method could be one of the existing methods or the new method. If there is uncertainty regarding parameter values, the new data-driven method would be a good choice. In summary, the numerical evaluations of the new data-driven methodology, presented in Section 6 and the supplementary material, show that the power of the data-driven method was close to the upper boundary of all the methods considered; i.e. it always performed relatively well. Also, the new data-driven method was always similar in performance to the more powerful of the two previously described methods. That is, it was either marginally more or less powerful, across all the scenarios tested, than the most powerful of either the Stallard or the Friede *et al.* methods. More specifically, when we assumed positive study findings for both early and final outcomes, the new method generally performed better than either the Stallard or the Friede *et al.* methods. Therefore, given that *a priori* we would generally not know which of the two previously described methods will perform the better, the new data-driven method is a good choice, as it will perform nearly as well or often better than either of the other methods.

The new data-driven method described here provides a straightforward and appealing way of choosing between two methods that have been suggested elsewhere for treatment selection in the chosen setting, where up to now there was no clear guidance. Our simulations have shown that this new method generally performs well. The new method just represents another way of using the data available at the interim analysis to select a treatment, and we accept that this is somewhat arbitrary. An interesting area for future work would be to attempt to make this decision in an optimal way, perhaps using prior knowledge of the expected magnitude of effects and associations between the endpoints in a Bayesian setting.

## Supporting Information

Supporting info item
