# Supplementary material for: Data-driven treatment selection for seamless phase II/III trials incorporating early-outcome data
Source: Pharm Stat. 2014 May 2;13(4):238–46. doi: 10.1002/pst.1619 (PMC4283755; doi:10.1002/pst.1619)
Supplement: Supplementary file 1 — Supporting info item [file pst0013-0238-sd1.pdf]

Supporting information for "Data-driven treatment selection for seamless phase II/III trials incorporating early-outcome datas"

Cornelia Ursula Kunz[a], Tim Friede[b], Nicholas Parsons[a], Susan Todd[c] and Nigel Stallard[a]

[a] Warwick Medical School, The University of Warwick, Coventry, CV4 7AL, U.K.

[b] Department of Medical Statistics, University Medical Center Göttingen,

[c] Department of Mathematics and Statistics, University of Reading, U.K.

1 Additional Figures

In addition to the results presented in the article, we investigated several other parameter settings. In each case, we assume that $\mu_{b_2} = 0.5\mu_{b_1}$, $\mu_{b_3} = 0.25\mu_{b_1}$, $\mu_{B_2} = 0.5\mu_{B_1}$, $\mu_{B_3} = 0.25\mu_{B_1}$, and $\sigma = \sigma_0 = 1$. The following figures show the results for:

Figure 1.1 $\mu_{b_1} = -0.2$, $n_1 = 4$, $N_1 = 32$, and $N_2 = 64$

Figure 1.2 $\mu_{b_1} = 0$, $n_1 = 4$, $N_1 = 32$, and $N_2 = 64$

Figure 1.3 $\mu_{b_1} = +0.2$, $n_1 = 4$, $N_1 = 32$, and $N_2 = 64$

Figure 1.4 $\mu_{b_1} = -0.2$, $n_1 = 8$, $N_1 = 32$, and $N_2 = 64$

Figure 1.5 $\mu_{b_1} = 0$, $n_1 = 8$, $N_1 = 32$, and $N_2 = 64$

Figure 1.6 $\mu_{b_1} = +0.2$, $n_1 = 8$, $N_1 = 32$, and $N_2 = 64$

Figure 1.7 $\mu_{b_1} = -0.2$, $n_1 = 16$, $N_1 = 32$, and $N_2 = 64$

Figure 1.8 $\mu_{b_1} = 0$, $n_1 = 16$, $N_1 = 32$, and $N_2 = 64$

Figure 1.9 $\mu_{b_1} = +0.2$, $n_1 = 16$, $N_1 = 32$, and $N_2 = 64$

Figure 1.10 $\mu_{b_1} = -0.2$, $n_1 = 4$, $N_1 = 64$, and $N_2 = 128$

Figure 1.11 $\mu_{b_1} = 0$, $n_1 = 4$, $N_1 = 64$, and $N_2 = 128$

Figure 1.12 $\mu_{b_1} = +0.2$, $n_1 = 4$, $N_1 = 64$, and $N_2 = 128$

Figure 1.13 $\mu_{b_1} = -0.2$, $n_1 = 8$, $N_1 = 64$, and $N_2 = 128$

Figure 1.14 $\mu_{b_1} = 0$, $n_1 = 8$, $N_1 = 64$, and $N_2 = 128$

Figure 1.15 $\mu_{b_1} = +0.2$, $n_1 = 8$, $N_1 = 64$, and $N_2 = 128$

Figure 1.16 $\mu_{b_1} = -0.2$, $n_1 = 16$, $N_1 = 64$, and $N_2 = 128$

Figure 1.17 $\mu_{b_1} = 0$, $n_1 = 16$, $N_1 = 64$, and $N_2 = 128$

Figure 1.18 $\mu_{b_1} = +0.2$, $n_1 = 16$, $N_1 = 64$, and $N_2 = 128$

For all figures results are given for $\rho_w = -0.9, -0.5, 0, 0.5, 0.9$ and μ_{B_1} from -0.5 to 1 in steps of 0.1.

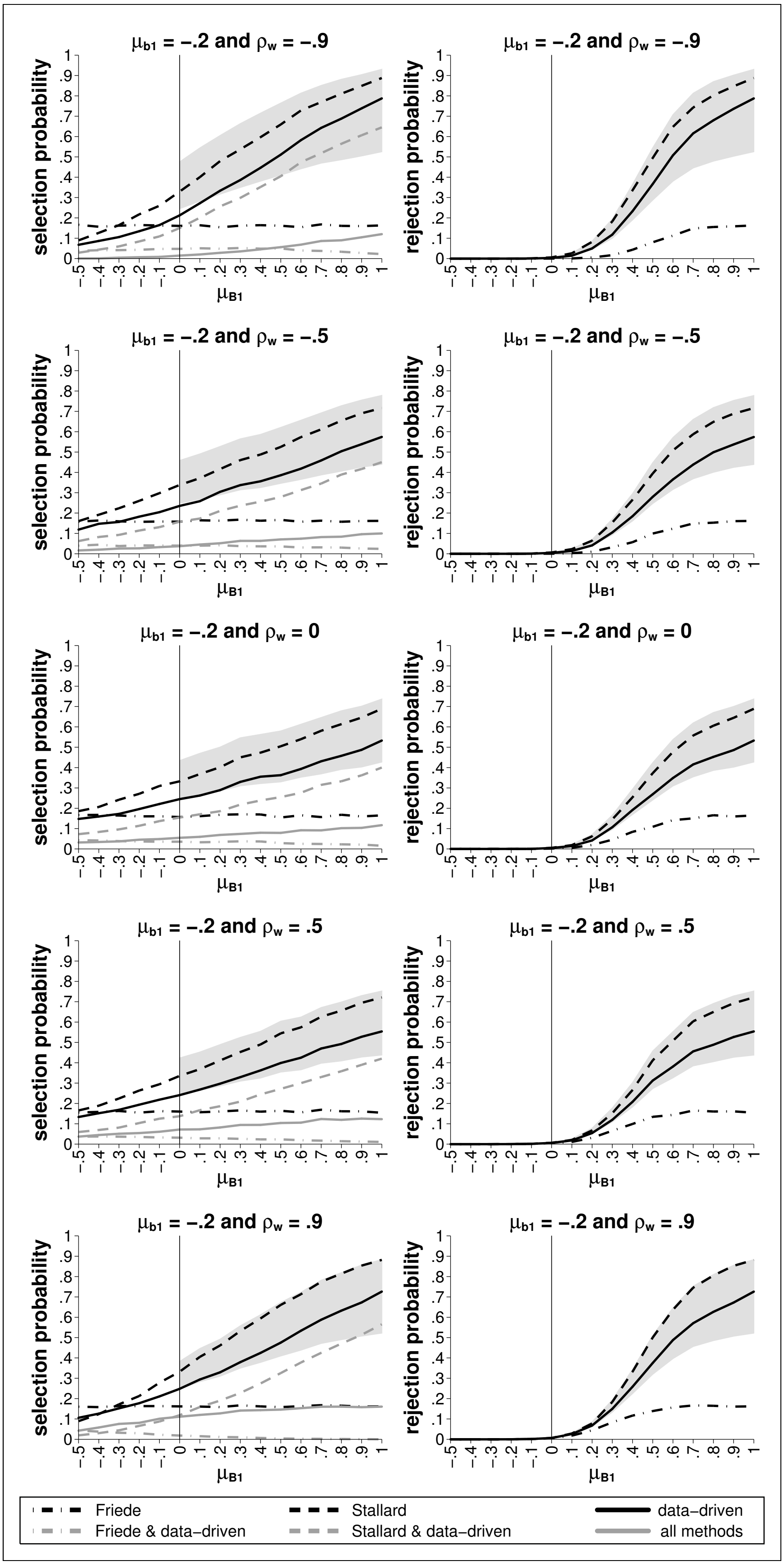

Figure 1.1: Selection and rejection probability for T_1 for different values of ρ_w and μ_{B_1} with $(\mu_{b_1}, \mu_{b_2}, \mu_{b_3}) = (-0.2, -0.1, -0.05)$, $\mu_{B_2} = 0.5\mu_{B_1}$, $\mu_{B_3} = 0.25\mu_{B_1}$, $n_1 = 4$, $N_1 = 32$, and $N_2 = 64$

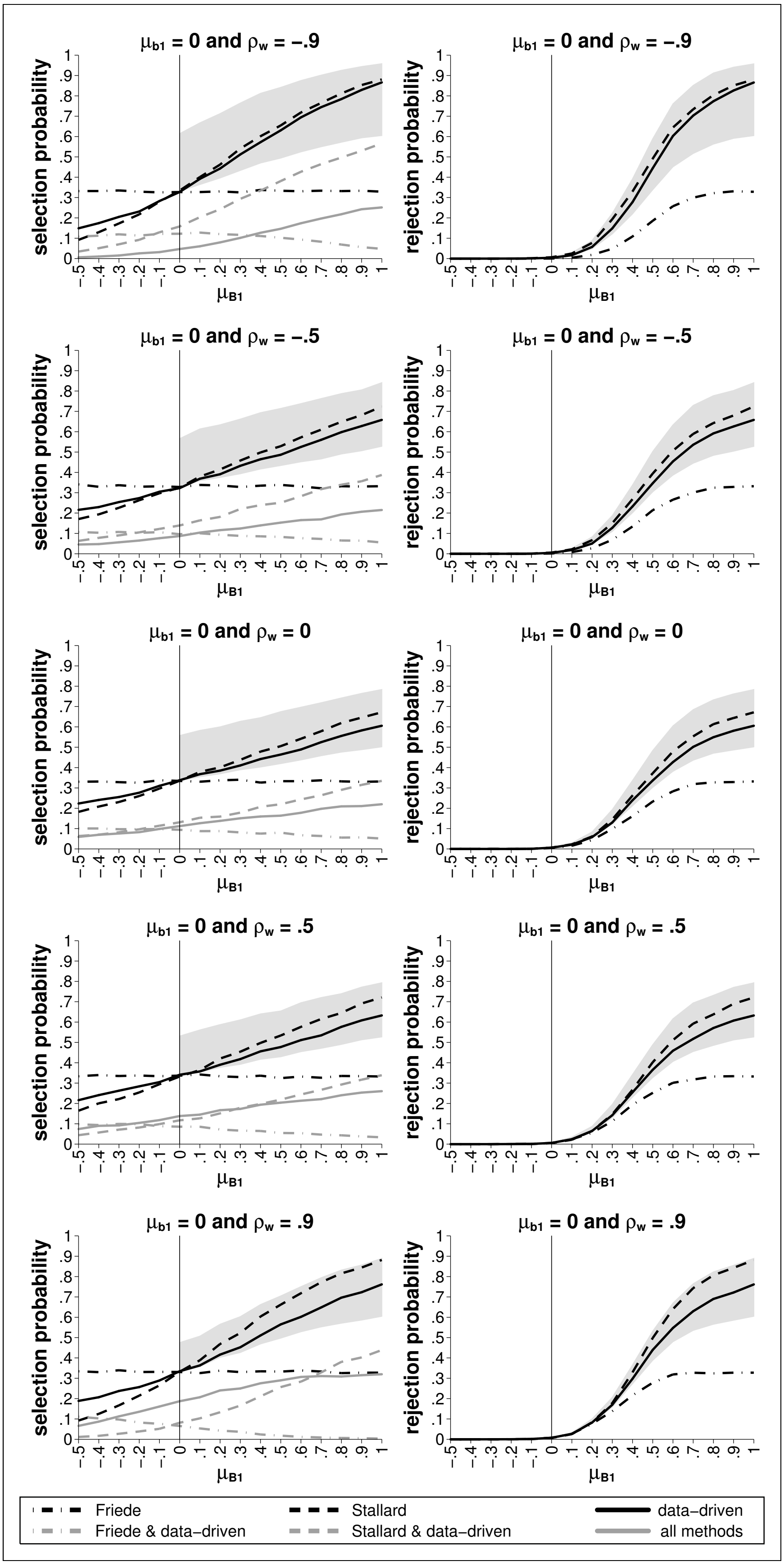

Figure 1.2: Selection and rejection probability for T_1 for different values of ρ_w and μ_{B_1} with $(\mu_{b_1}, \mu_{b_2}, \mu_{b_3}) = (0, 0, 0)$, $\mu_{B_2} = 0.5\mu_{B_1}$, $\mu_{B_3} = 0.25\mu_{B_1}$, $n_1 = 4$, $N_1 = 32$, and $N_2 = 64$

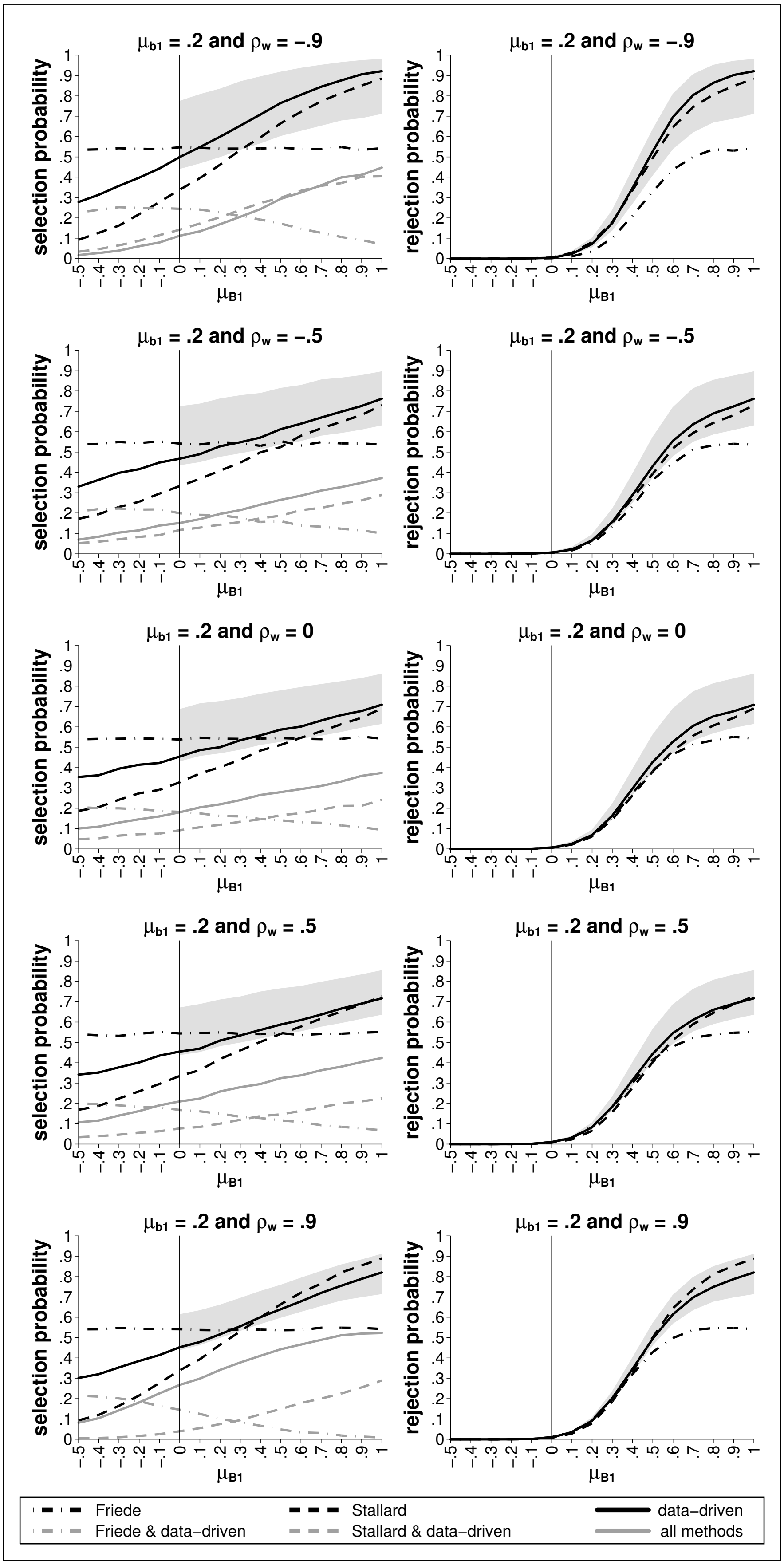

Figure 1.3: Selection and rejection probability for T_1 for different values of ρ_w and μ_{B_1} with $(\mu_{b_1}, \mu_{b_2}, \mu_{b_3}) = (0.2, 0.1, 0.05)$, $\mu_{B_2} = 0.5\mu_{B_1}$, $\mu_{B_3} = 0.25\mu_{B_1}$, $n_1 = 4$, $N_1 = 32$, and $N_2 = 64$

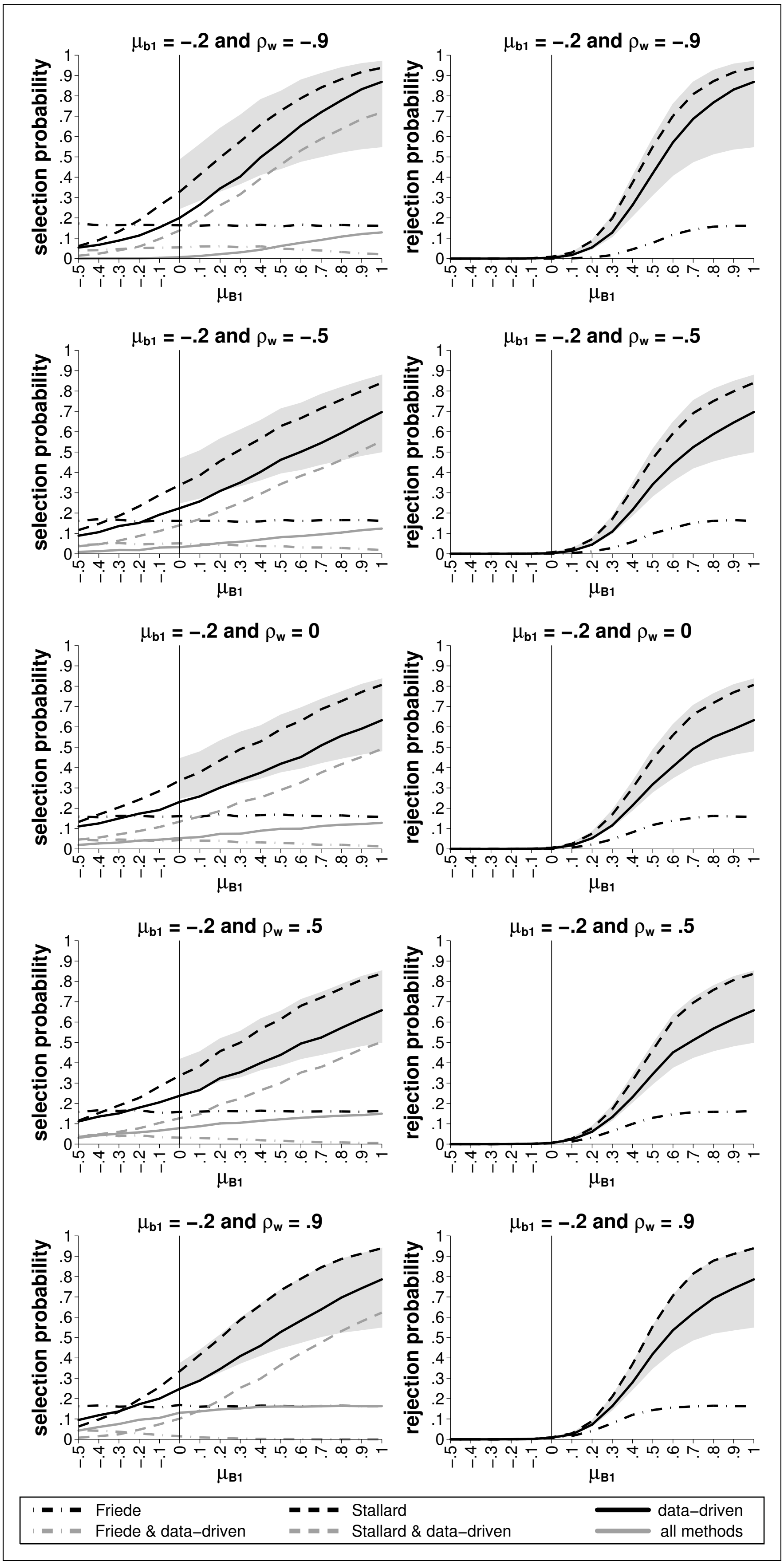

Figure 1.4: Selection and rejection probability for T_1 for different values of ρ_w and μ_{B_1} with $(\mu_{b_1}, \mu_{b_2}, \mu_{b_3}) = (-0.2, -0.1, -0.05)$, $\mu_{B_2} = 0.5\mu_{B_1}$, $\mu_{B_3} = 0.25\mu_{B_1}$, $n_1 = 8$, $N_1 = 32$, and $N_2 = 64$

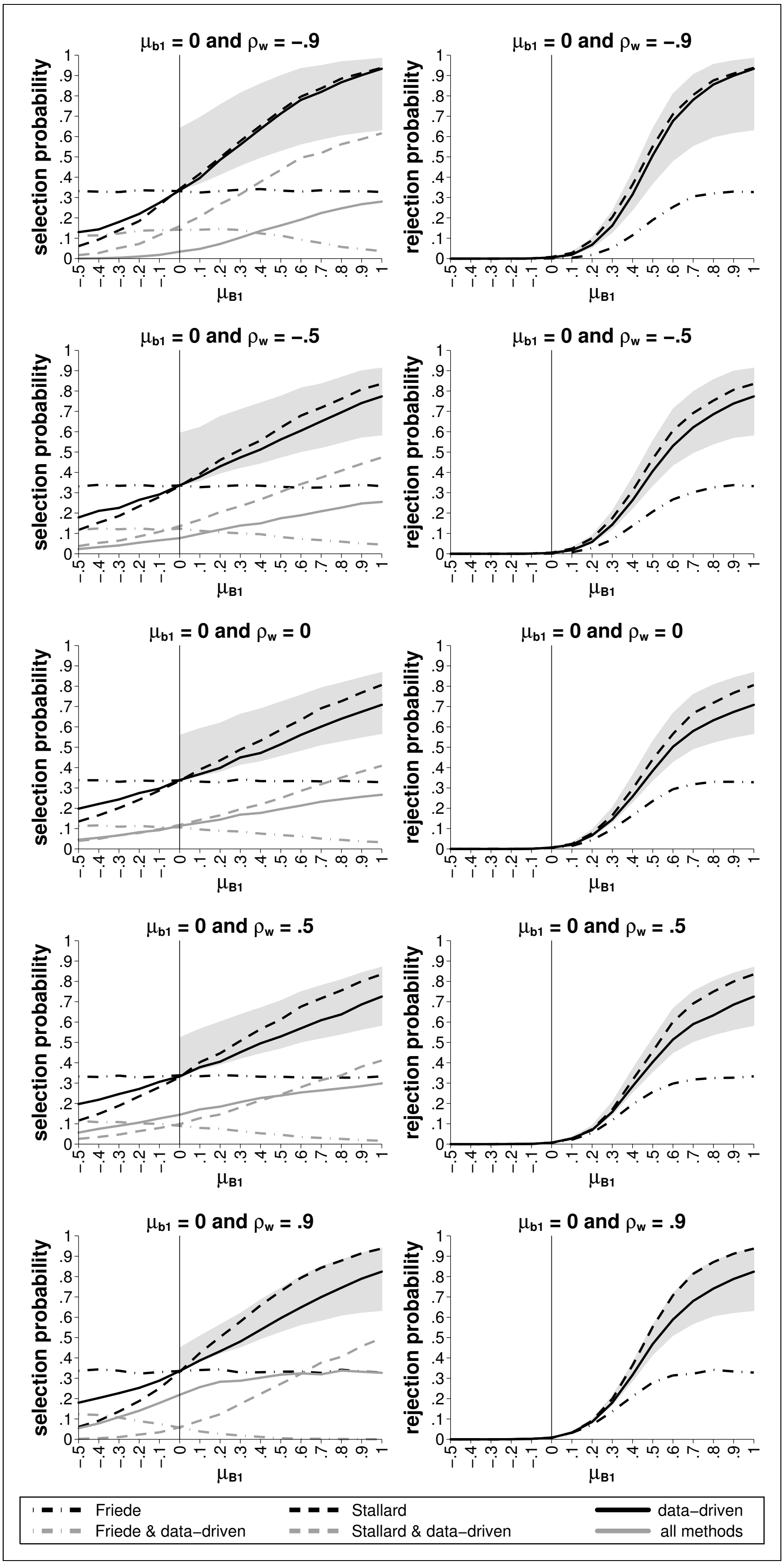

Figure 1.5: Selection and rejection probability for T_1 for different values of ρ_w and μ_{B_1} with $(\mu_{b_1}, \mu_{b_2}, \mu_{b_3}) = (0, 0, 0)$, $\mu_{B_2} = 0.5\mu_{B_1}$, $\mu_{B_3} = 0.25\mu_{B_1}$, $n_1 = 8$, $N_1 = 32$, and $N_2 = 64$

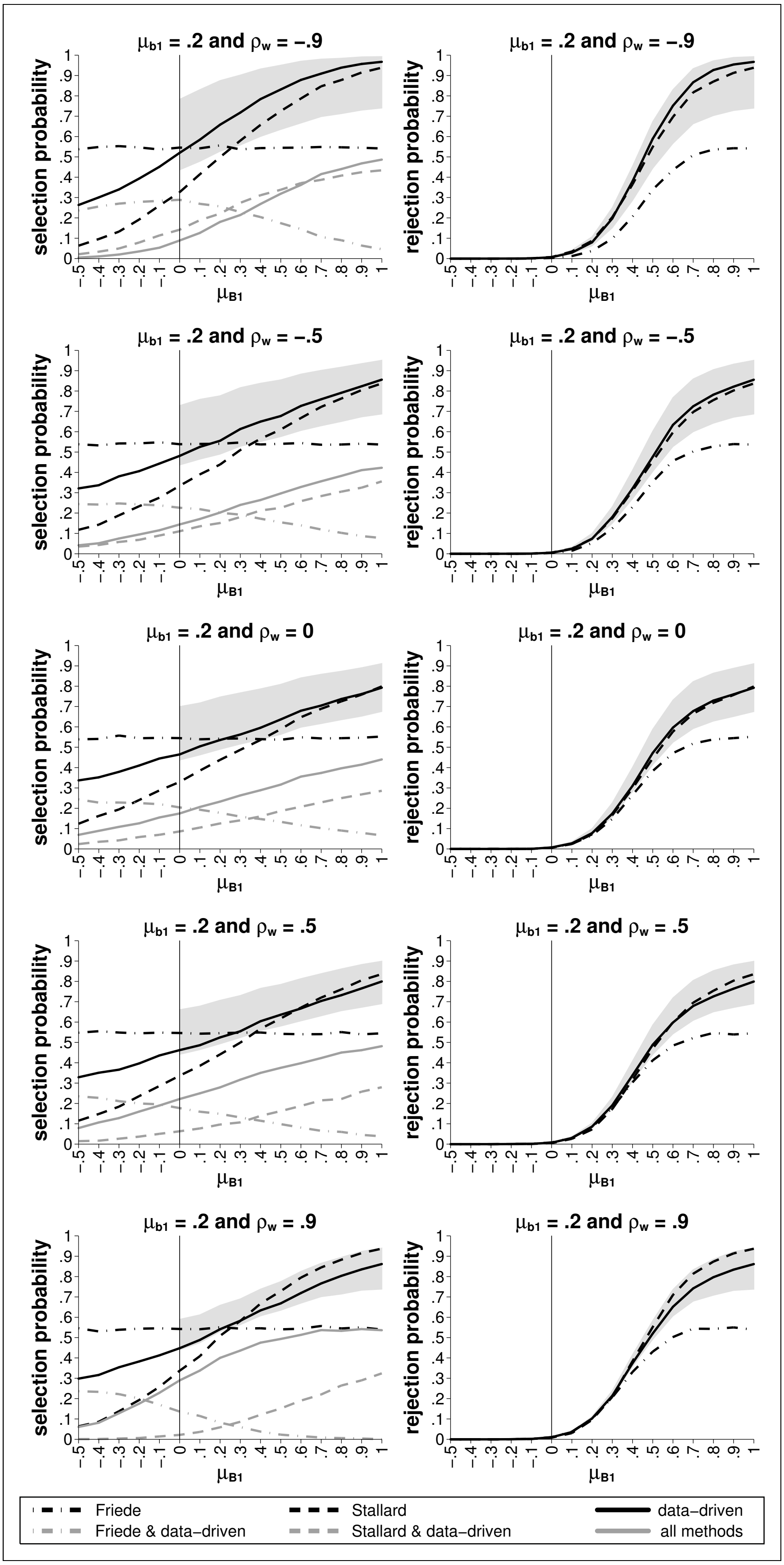

Figure 1.6: Selection and rejection probability for T_1 for different values of ρ_w and μ_{B_1} with $(\mu_{b_1}, \mu_{b_2}, \mu_{b_3}) = (0.2, 0.1, 0.05)$, $\mu_{B_2} = 0.5\mu_{B_1}$, $\mu_{B_3} = 0.25\mu_{B_1}$, $n_1 = 8$, $N_1 = 32$, and $N_2 = 64$

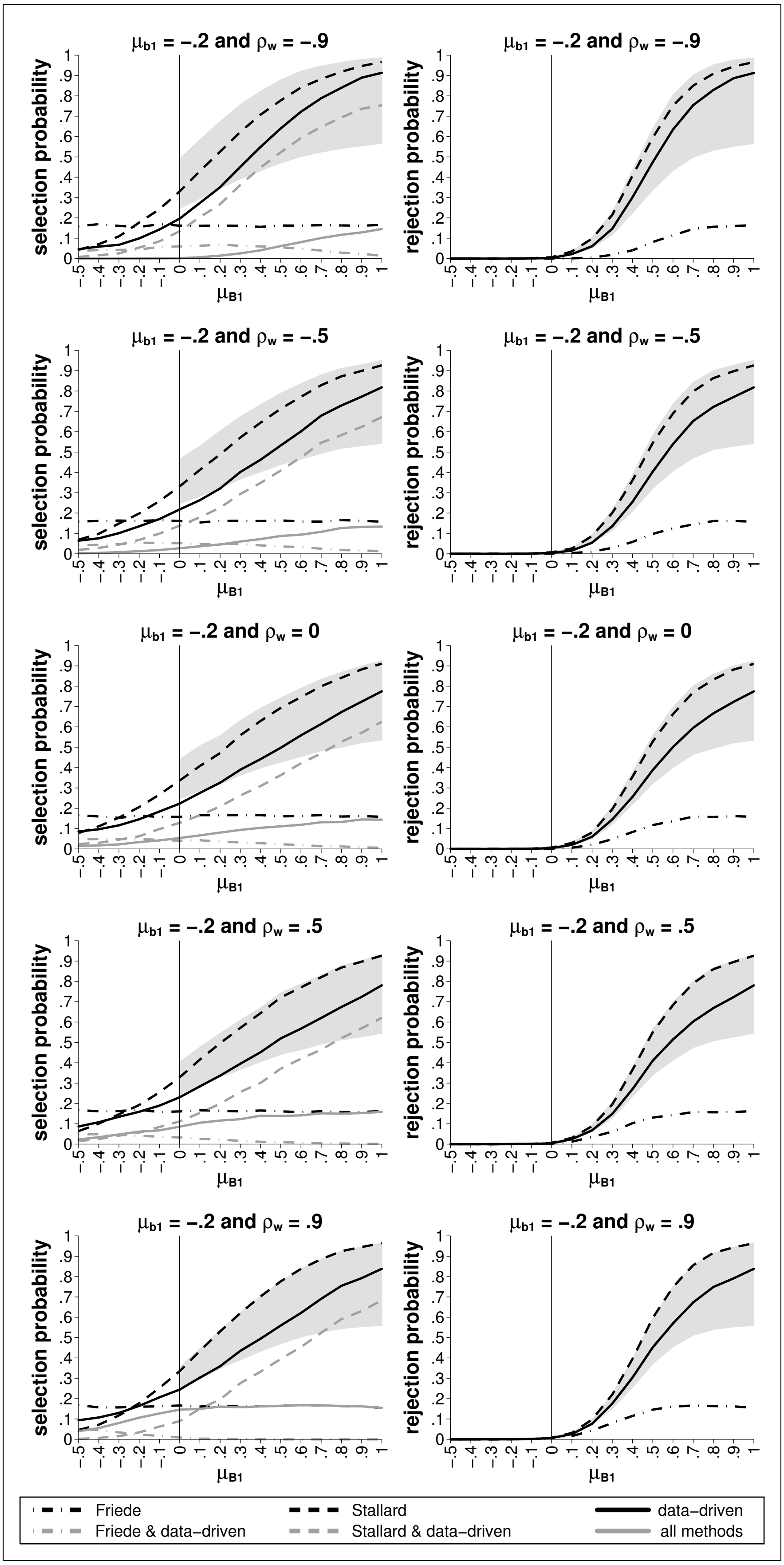

Figure 1.7: Selection and rejection probability for T_1 for different values of ρ_w and μ_{B_1} with $(\mu_{b_1}, \mu_{b_2}, \mu_{b_3}) = (-0.2, -0.1, -0.05)$, $\mu_{B_2} = 0.5\mu_{B_1}$, $\mu_{B_3} = 0.25\mu_{B_1}$, $n_1 = 16$, $N_1 = 32$, and $N_2 = 64$

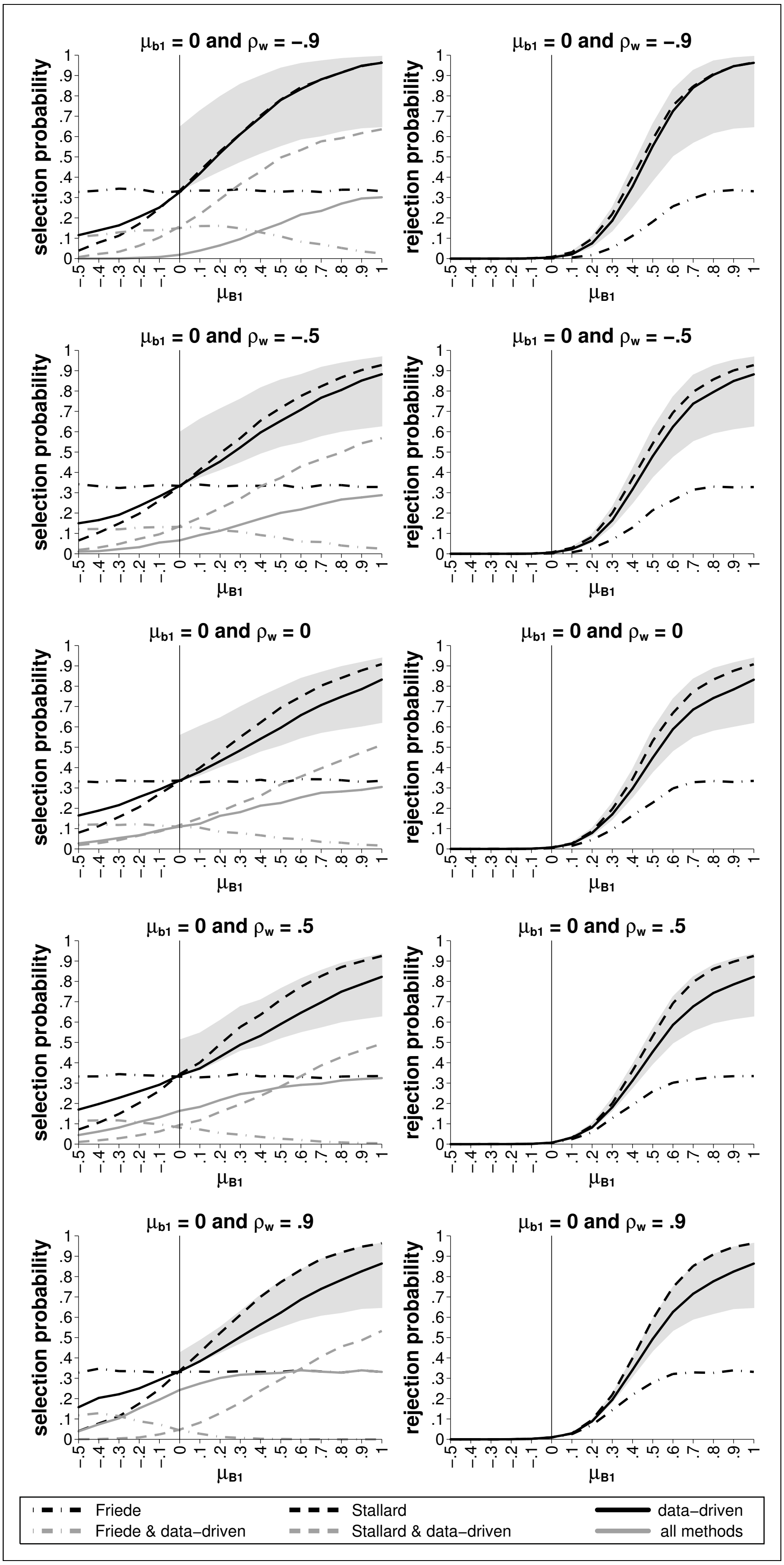

Figure 1.8: Selection and rejection probability for T_1 for different values of ρ_w and μ_{B_1} with $(\mu_{b_1}, \mu_{b_2}, \mu_{b_3}) = (0, 0, 0)$, $\mu_{B_2} = 0.5\mu_{B_1}$, $\mu_{B_3} = 0.25\mu_{B_1}$, $n_1 = 16$, $N_1 = 32$, and $N_2 = 64$

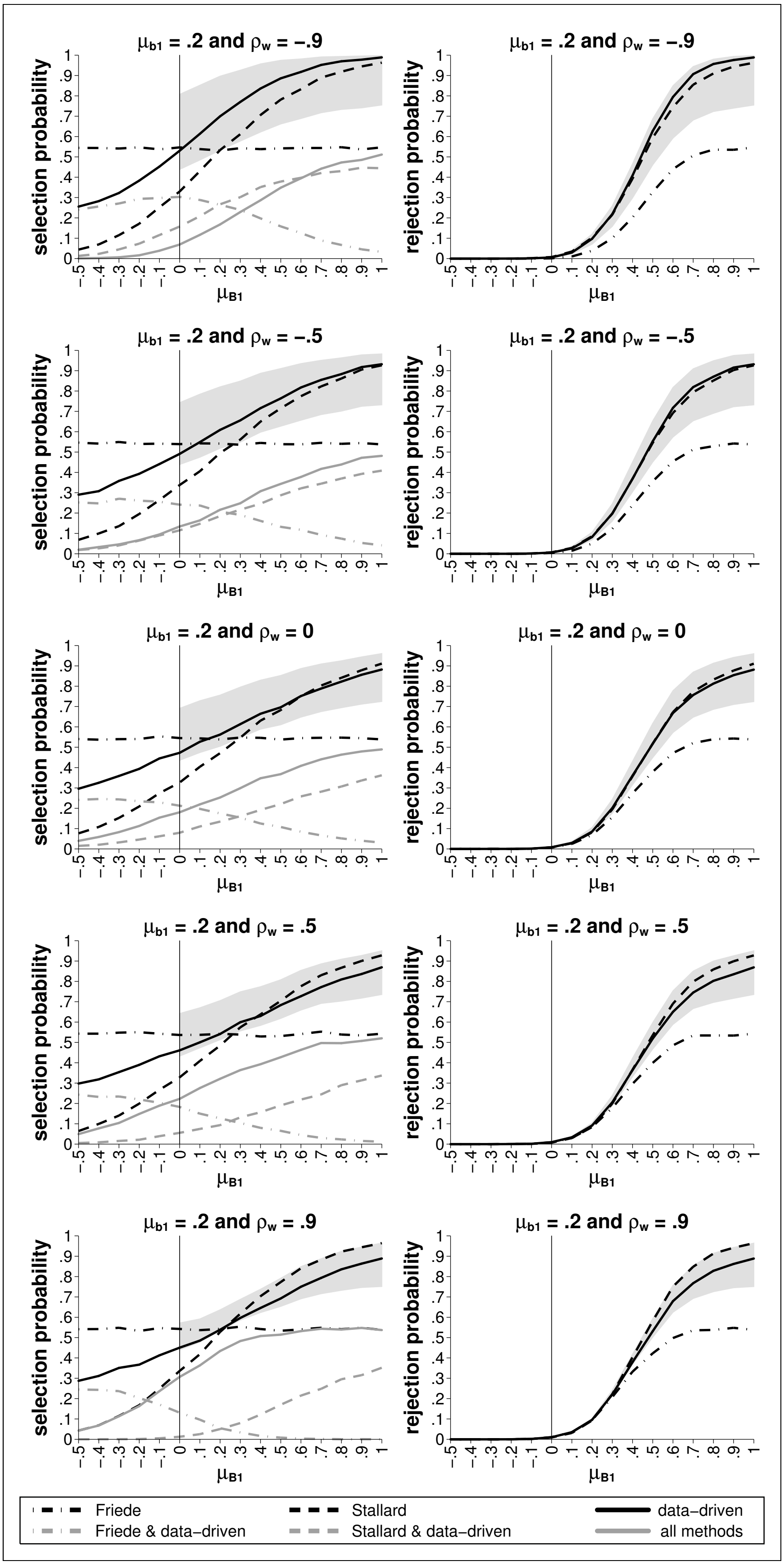

Figure 1.9: Selection and rejection probability for T_1 for different values of ρ_w and μ_{B_1} with $(\mu_{b_1}, \mu_{b_2}, \mu_{b_3}) = (0.2, 0.1, 0.05)$, $\mu_{B_2} = 0.5\mu_{B_1}$, $\mu_{B_3} = 0.25\mu_{B_1}$, $n_1 = 16$, $N_1 = 32$, and $N_2 = 64$

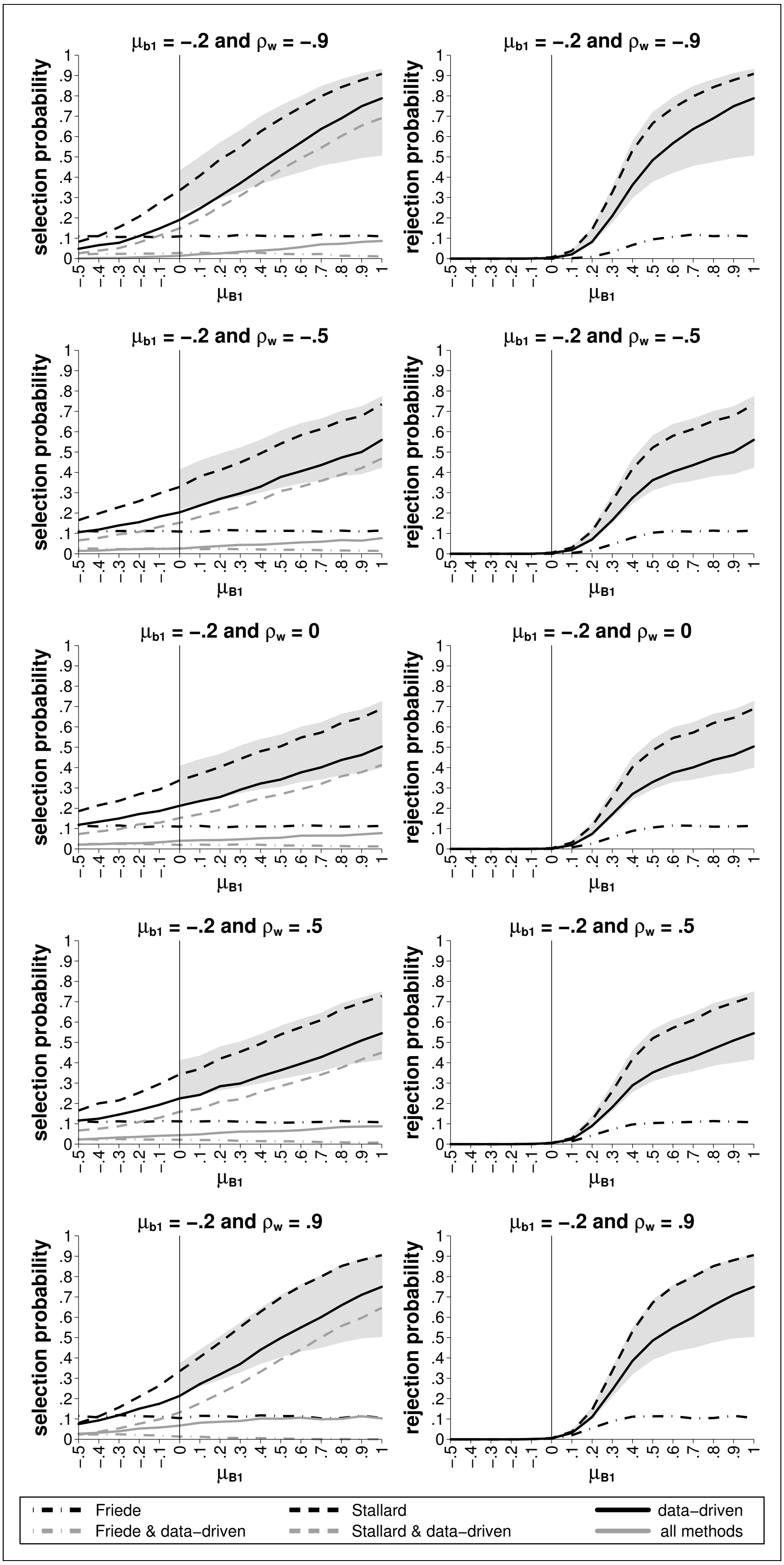

Figure 1.10: Selection and rejection probability for T_1 for different values of ρ_w and μ_{B_1} with $(\mu_{b_1}, \mu_{b_2}, \mu_{b_3}) = (-0.2, -0.1, -0.05)$, $\mu_{B_2} = 0.5\mu_{B_1}$, $\mu_{B_3} = 0.25\mu_{B_1}$, $n_1 = 4$, $N_1 = 64$, and $N_2 = 128$

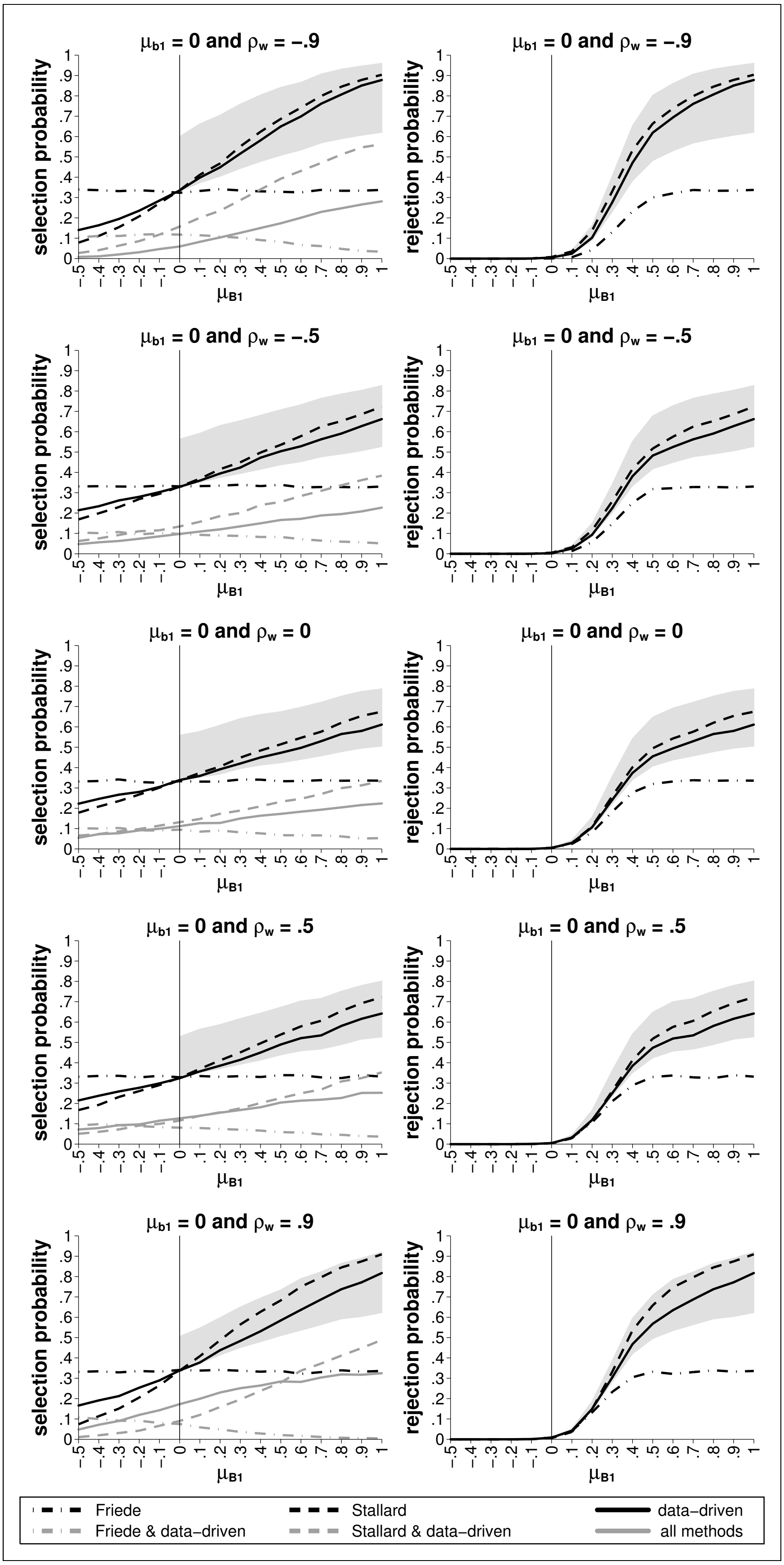

Figure 1.11: Selection and rejection probability for T_1 for different values of ρ_w and μ_{B_1} with $(\mu_{b_1}, \mu_{b_2}, \mu_{b_3}) = (0, 0, 0)$, $\mu_{B_2} = 0.5\mu_{B_1}$, $\mu_{B_3} = 0.25\mu_{B_1}$, $n_1 = 4$, $N_1 = 64$, and $N_2 = 128$

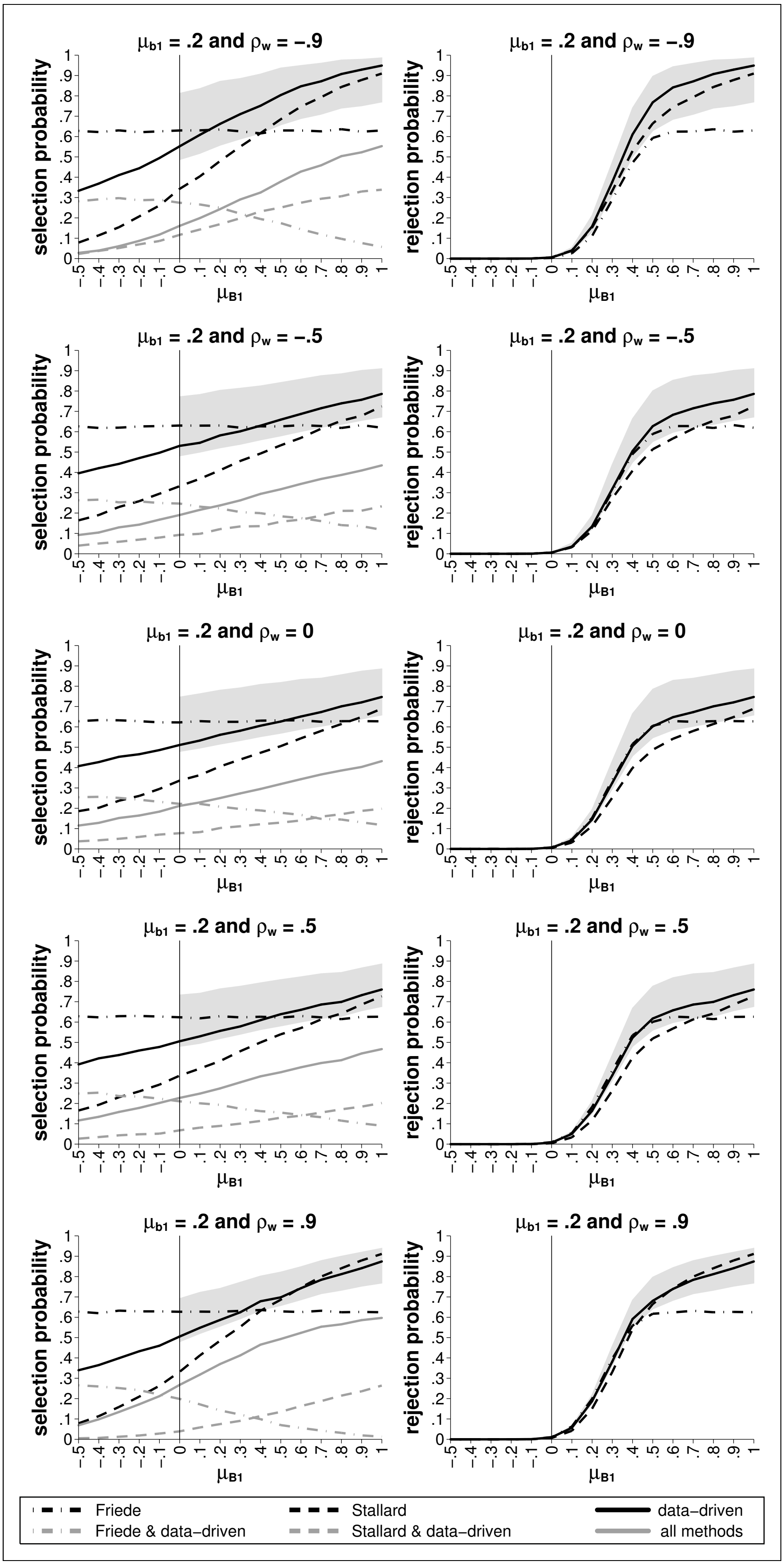

Figure 1.12: Selection and rejection probability for T_1 for different values of ρ_w and μ_{B_1} with $(\mu_{b_1}, \mu_{b_2}, \mu_{b_3}) = (0.2, 0.1, 0.05)$, $\mu_{B_2} = 0.5\mu_{B_1}$, $\mu_{B_3} = 0.25\mu_{B_1}$, $n_1 = 4$, $N_1 = 64$, and $N_2 = 128$

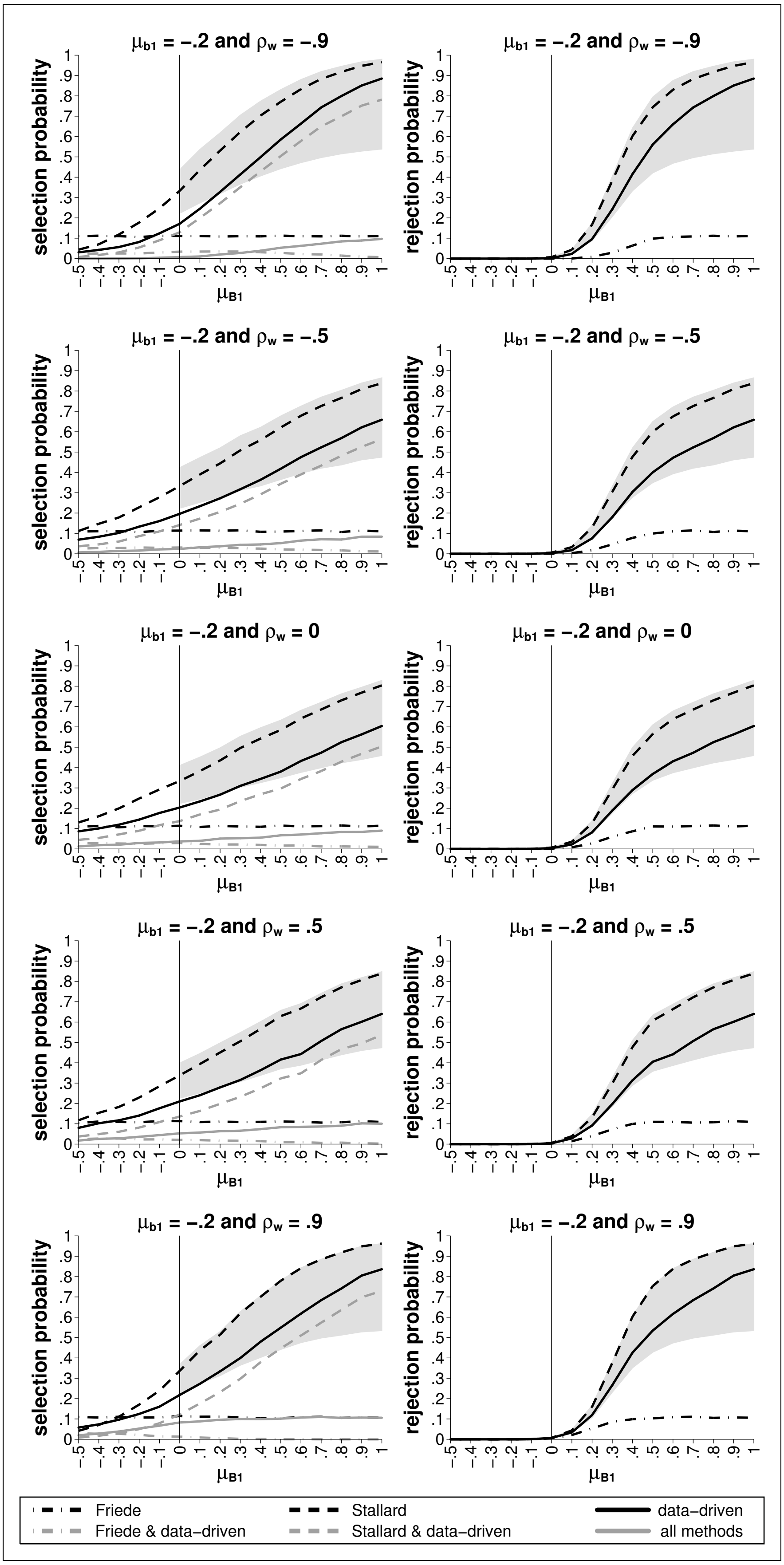

Figure 1.13: Selection and rejection probability for T_1 for different values of ρ_w and μ_{B_1} with $(\mu_{b_1}, \mu_{b_2}, \mu_{b_3}) = (-0.2, -0.1, -0.05)$, $\mu_{B_2} = 0.5\mu_{B_1}$, $\mu_{B_3} = 0.25\mu_{B_1}$, $n_1 = 8$, $N_1 = 64$, and $N_2 = 128$

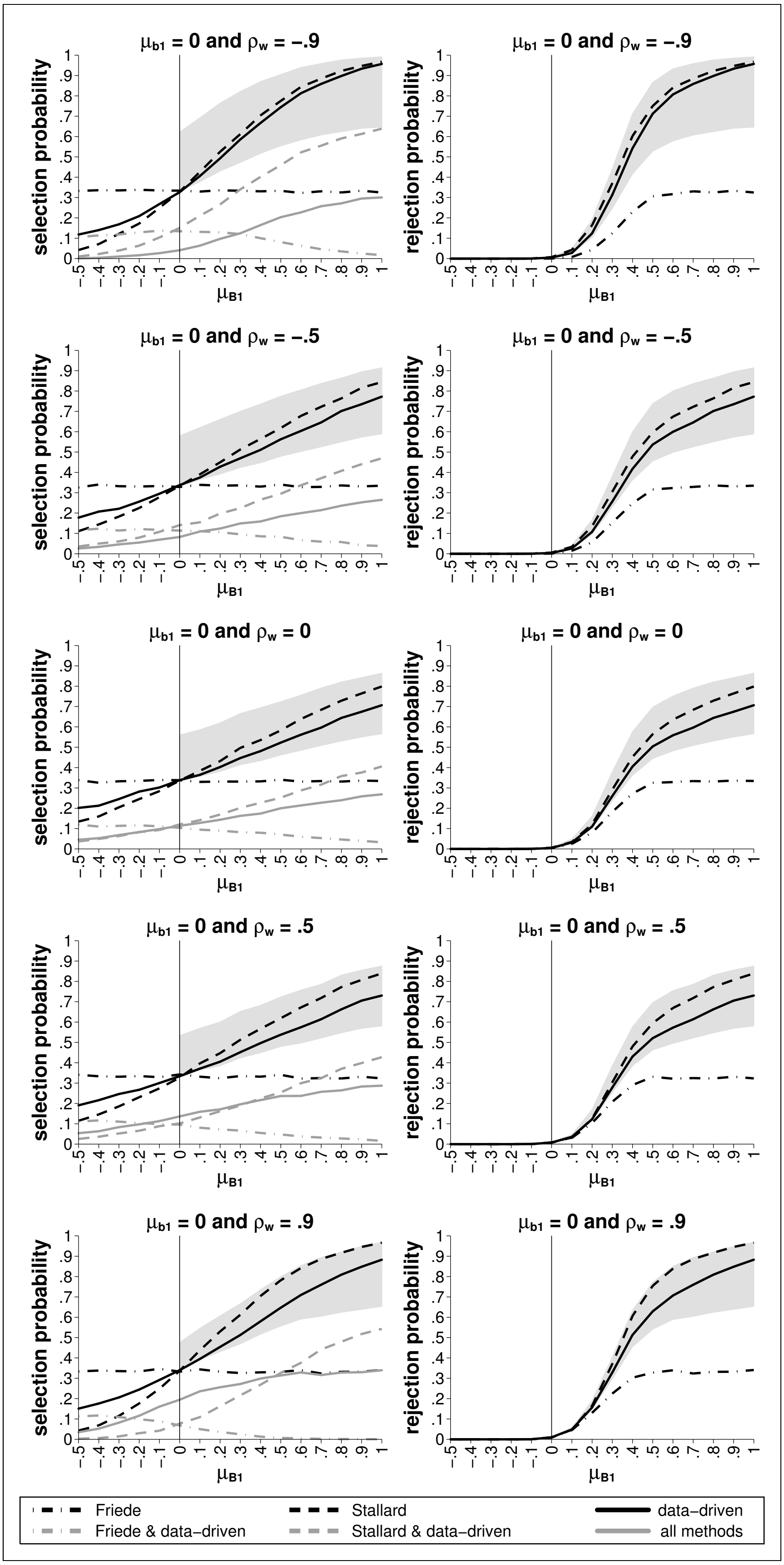

Figure 1.14: Selection and rejection probability for T_1 for different values of ρ_w and μ_{B_1} with $(\mu_{b_1}, \mu_{b_2}, \mu_{b_3}) = (0, 0, 0)$, $\mu_{B_2} = 0.5\mu_{B_1}$, $\mu_{B_3} = 0.25\mu_{B_1}$, $n_1 = 8$, $N_1 = 64$, and $N_2 = 128$

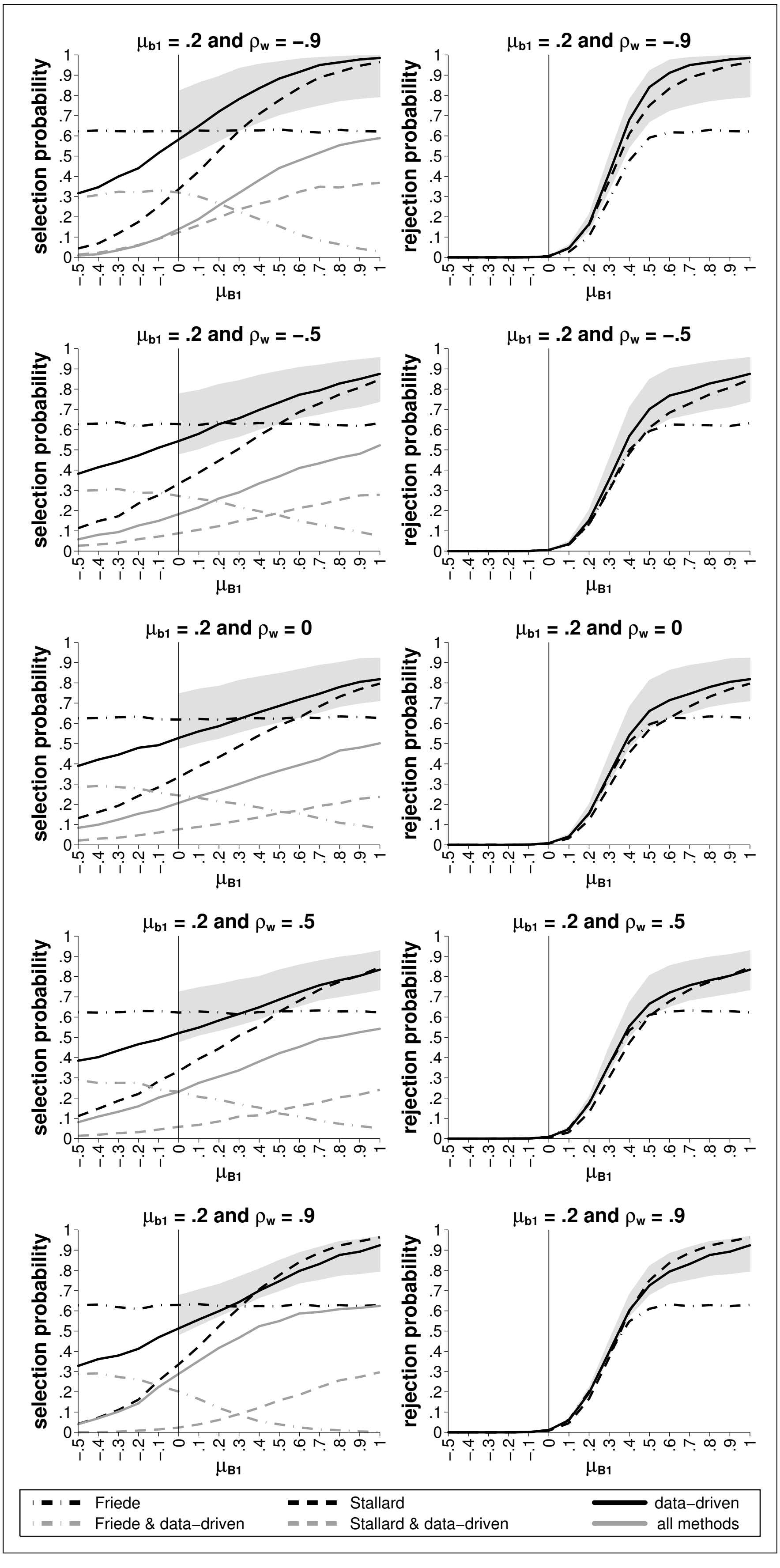

Figure 1.15: Selection and rejection probability for T_1 for different values of ρ_w and μ_{B_1} with $(\mu_{b_1}, \mu_{b_2}, \mu_{b_3}) = (0.2, 0.1, 0.05)$, $\mu_{B_2} = 0.5\mu_{B_1}$, $\mu_{B_3} = 0.25\mu_{B_1}$, $n_1 = 8$, $N_1 = 64$, and $N_2 = 128$

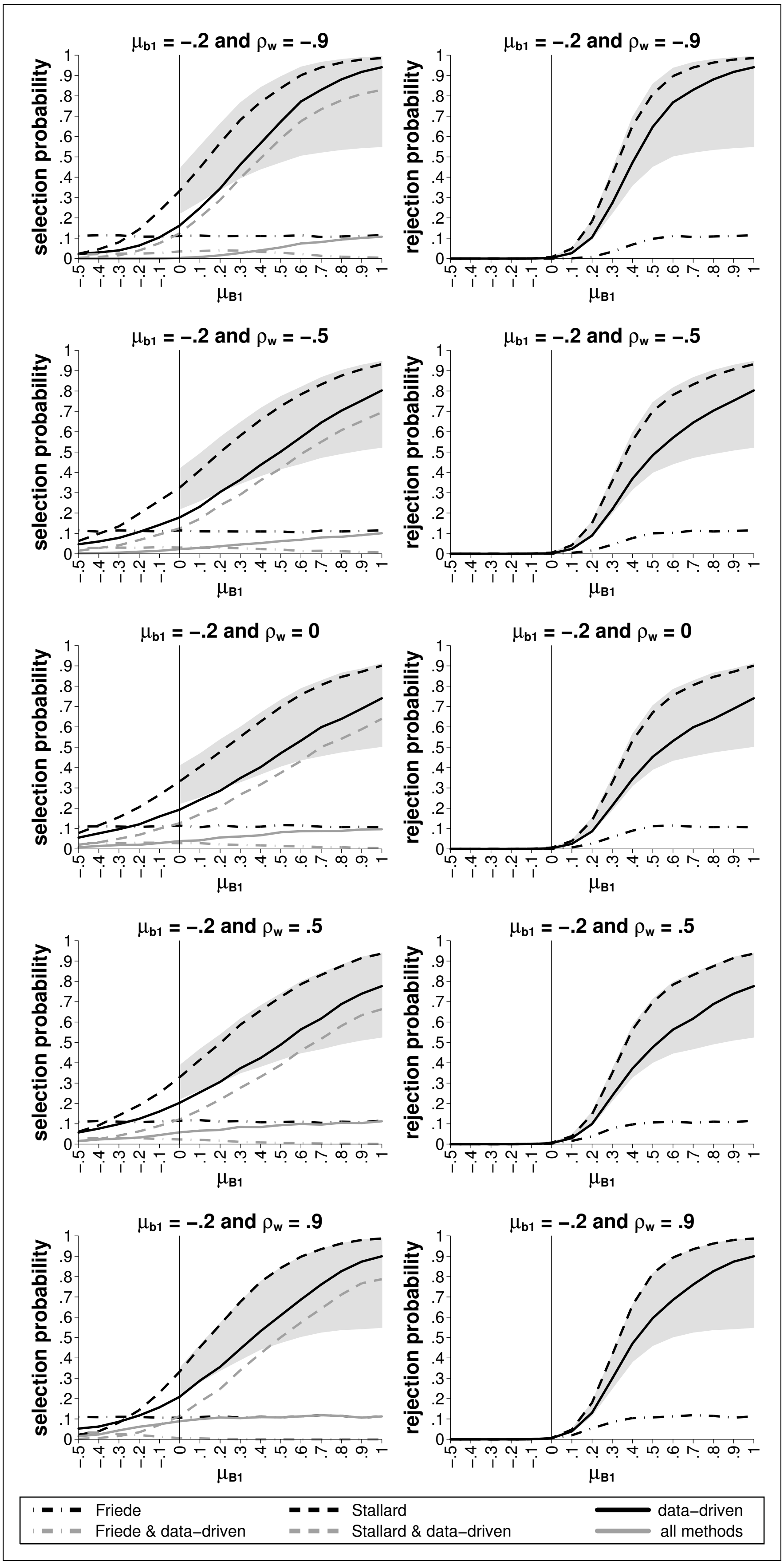

Figure 1.16: Selection and rejection probability for T_1 for different values of ρ_w and μ_{B_1} with $(\mu_{b_1}, \mu_{b_2}, \mu_{b_3}) = (-0.2, -0.1, -0.05)$, $\mu_{B_2} = 0.5\mu_{B_1}$, $\mu_{B_3} = 0.25\mu_{B_1}$, $n_1 = 16$, $N_1 = 64$, and $N_2 = 128$

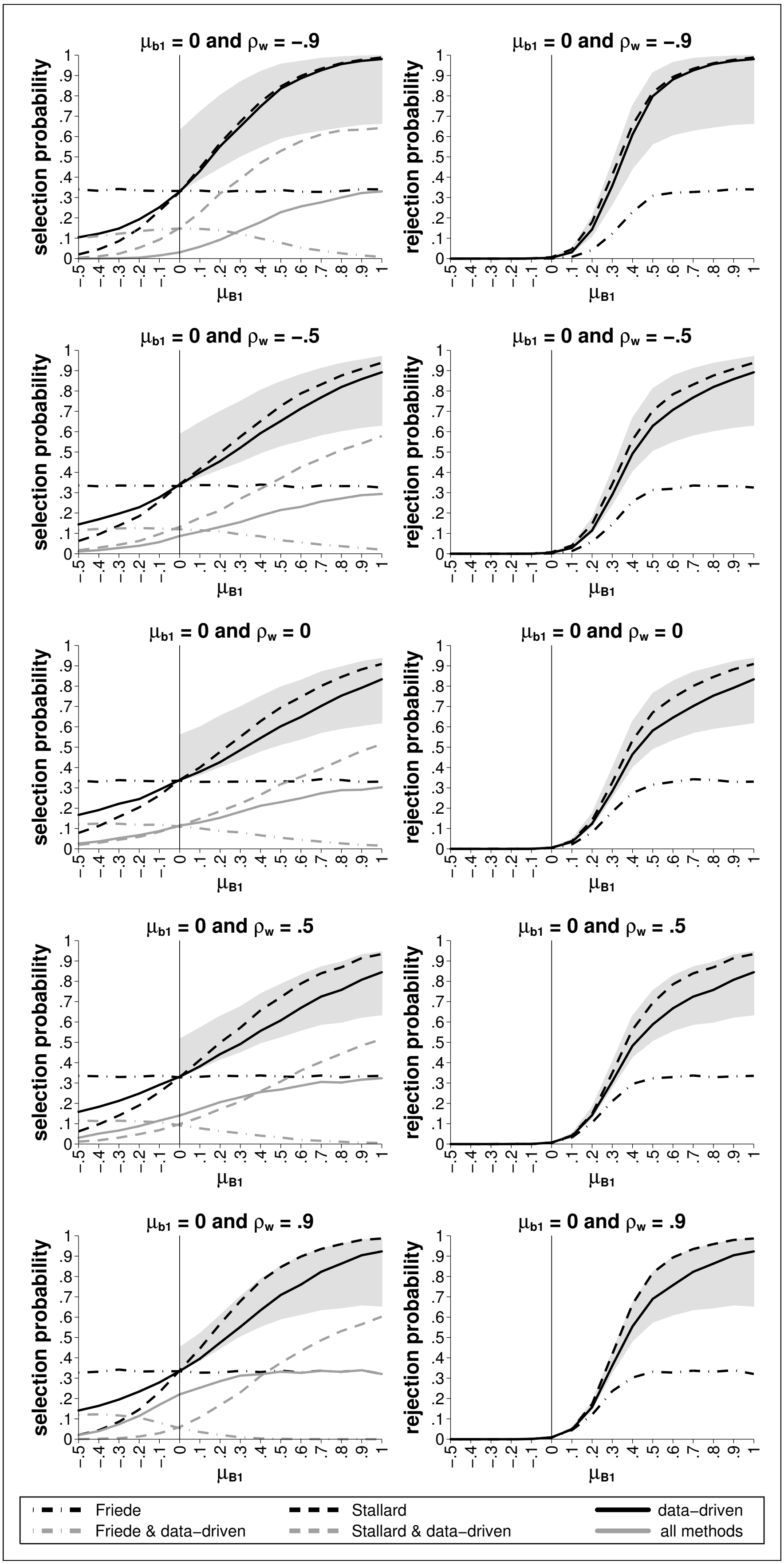

Figure 1.17: Selection and rejection probability for T_1 for different values of ρ_w and μ_{B_1} with $(\mu_{b_1}, \mu_{b_2}, \mu_{b_3}) = (0, 0, 0)$, $\mu_{B_2} = 0.5\mu_{B_1}$, $\mu_{B_3} = 0.25\mu_{B_1}$, $n_1 = 16$, $N_1 = 64$, and $N_2 = 128$

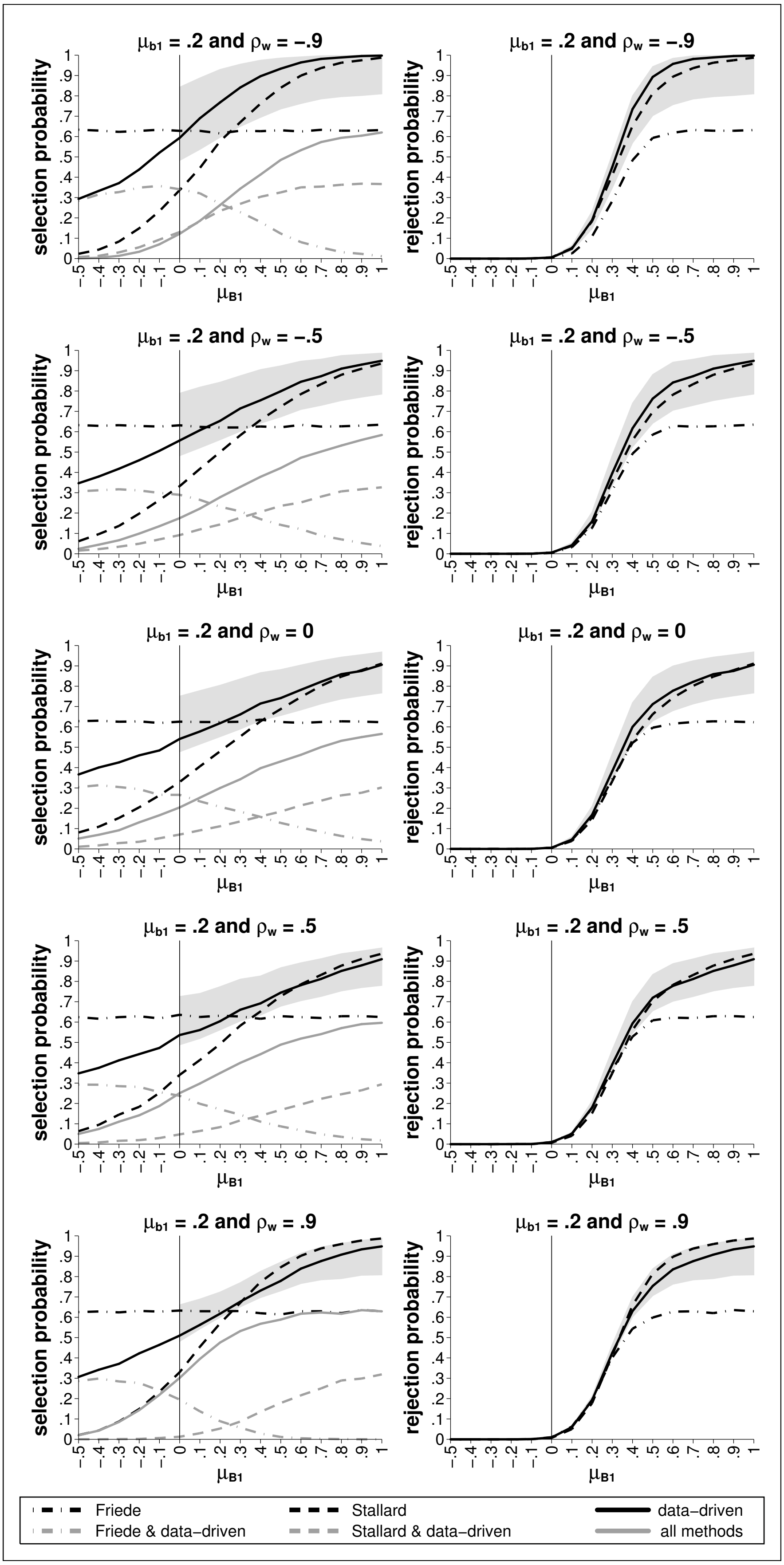

Figure 1.18: Selection and rejection probability for T_1 for different values of ρ_w and μ_{B_1} with $(\mu_{b_1}, \mu_{b_2}, \mu_{b_3}) = (0.2, 0.1, 0.05)$, $\mu_{B_2} = 0.5\mu_{B_1}$, $\mu_{B_3} = 0.25\mu_{B_1}$, $n_1 = 16$, $N_1 = 64$, and $N_2 = 128$
